# Hydrological connectivity shape the nitrogen pollution sources and microbial community structure in a river-lake connected system

**DOI:** 10.3389/fmicb.2025.1563578

**Published:** 2025-04-11

**Authors:** Haoda Chen, Lulu Zhang, Zishuai Zheng, Yuang Gao, Yu Zhao

**Affiliations:** ^1^College of Environment Science and Engineering, Hebei University of Science and Technology, Shijiazhuang, Hebei, China; ^2^Biotechnology Laboratory for Pollution Control in Hebei, Shijiazhuang, China; ^3^State Key Laboratory of Environmental Aquatic Chemistry, Research Center for Eco-Environmental Sciences, Chinese Academy of Science, Beijing, China

**Keywords:** nitrogen pollution, microbial community structure, spatio-temporal distribution, river-lake connectivity, river-lake systems

## Abstract

Intensified agricultural and urban activities have exacerbated nitrogen pollution, posing a severe threat to freshwater ecosystems, particularly under intensified agricultural and urbanization activities. This study systematically examined Baiyangdian Lake (BYD) and its principal inflowing rivers, namely Fu River (FH), Baigouyin River (BGY), and Xiaoyi River (XY) to characterize the spatio-temporal distribution, primary nitrogen sources, and the impact on sediment microbial community structure. Results indicated pronounced seasonal variations in both nitrogen pollution loads and sources, with riverine nitrogen levels rising markedly from dry season (May) to wet season (August). Atmospheric deposition accounted for 43.9% of the nitrogen input dry season, whereas in wet season, agricultural fertilizers and sewage contributed 23.3 and 26.4%, respectively. Additionally, microbial communities exhibited distinct temporal and spatial patterns, with significantly higher diversity and species richness being during the wet season. The, microbial composition shifted, as evidenced by a decline in *Proteobacteria* and increases in *Firmicutes* and *Actinobacteriota*. River-lake connectivity emerged as a critical factor, with FH displaying a notably higher connectivity index in wet season compared to BGY and XY rivers. Structural equation modeling (SEM) analysis further revealed that river-lake connectivity was significantly and positively correlated with nitrogen pollution, was significantly and negatively correlated with microbial α-diversity. These findings demonstrated that river-lake connectivity directly influenced nitrogen concentrations, which in turn indirectly modulated microbial diversity.

## 1 Introduction

The influence of human activities on environmental quality has intensified significantly in recent decades. Industrial, agricultural, aquaculture and domestic wastewater discharge large amounts of nitrogen-containing compounds into aquatic ecosystems (Rudneva and Omel'chenko, 2021). It is estimated that ~120 million tons of reactive nitrogen are discharged into water bodies annually in China. In aquatic ecosystems, nitrogen pollution can lead to the deterioration of water quality, eutrophication, algal blooms, and a decrease in oxygen availability for aquatic organisms (Yu et al., 2019; Qian et al., 2025). This is especially true for microbial communities, which play a crucial role in nitrogen cycling processes, such as nitrogen fixation, nitrification, denitrification, and anaerobic ammonia oxidation (Wen M. et al., 2024). Therefore, nitrogen pollution has become an important environmental problem, that has attracted wide attention from researchers worldwide (Wang M. et al., 2024). Previous studies have focused on the nitrogen pollution source analysis, nitrogen transformation processes, and their impacts on aquatic ecosystems (Wang J. et al., 2024; Xu et al., 2024; Zhou et al., 2024). However, most existing studies examine nitrogen pollution and cycling processes at broader watershed scales, yet they often overlook the influence of connectivity on the spatial and temporal variations of nitrogen dynamics. As a result, a comprehensive understanding of how hydrological connectivity regulates nitrogen pollution sources and microbial community structure in river-lake systems is still lacking.

Currently, stable isotope tracing and the Soil and Water Assessment Tool (SWAT) model have been widely used for nitrogen pollution source identification, and each method has its own advantages and disadvantages (Guo et al., 2024; Arshad et al., 2025). In particular, nitrate (${\text{NO}}_{3}^{-}$-N) stable isotope analysis can precisely identify nitrogen pollution sources, such as soil nitrogen, atmospheric deposition, chemical fertilizers, and manure and sewage. For example, in the Pearl River Basin, agricultural runoff accounted for 52.25% of total nitrogen inputs during the rainy season, while urban wastewater increased significantly to 44.37% during the dry season based on ${\text{NO}}_{3}^{-}$-N stable isotope analysis (Wang C. et al., 2024). However, stable isotope analysis is more suitable for small-scale source identification and lacks the capacity to capture the pollution loads at a large scale. In view of this, the SWAT model can integrate various data including land use, hydrology, climate, and soil properties, to simulate nitrogen transport and transformation at the watershed scale (Arshad et al., 2025). For instance, the SWAT model was applied in the Mississippi River Basin to quantify the contributions of agricultural activities to nitrogen export and to assess the effectiveness of various mitigation measures (Forgrave et al., 2024). The SWAT model demonstrated an advantage in identifying the spatial and temporal patterns of pollution loads, with its precision depending on high-resolution input data at the sub-basin scale. Therefore, combining these two methods can integrate their individual advantages. The integrated application of stable isotope analysis and SWAT model simulations can be used to study how hydrological connectivity shapes nitrogen pollution sources and microbial community structure in a river-lake connected system.

Nitrogen pollution can be categorized into non-point source (NPS) pollution and point-source (PS) pollution based on its transport pathways. NPSs primarily enter rivers through soil erosion and leaching, while PSs pollution is directly discharged into water bodies via pipeline networks (Wen Y. et al., 2024). At the watershed scale, these two pollution sources exhibit significant differences in hydrological, physicochemical, and biological conditions, leading to distinct spatiotemporal variations in nitrogen pollution at watersheds scale. For example, in the Yellow River Basin, NPS pollution was the main nitrogen pollution source in the watershed, contributing up to ~350,000 tons/year through soil erosion (Zhang et al., 2024). In contrast, PS is generally associated with higher nitrogen concentrations, which strongly impact local microbial communities. For instance, in the Sava River Basin, PS pollution was the main nitrogen pollution source in water, particularly during the dry season, accounting for more than 60% of total nitrogen inputs (Vrzel et al., 2016). Hence, nitrogen source pollution is characterized by significant temporal and spatial differences. Moreover, long-term nitrogen inputs gradually alter microbial community structure and function in the aquatic ecosystem, promoting the proliferation of specific functional microbes (e.g., ammonia-oxidizing bacteria and denitrifiers) and affecting microbial functional diversity (Oulehle et al., 2015). In a river-lake connected system, the hydrological connectivity plays a crucial role in nitrogen migration and distribution at the watershed scale, thereby significantly affecting microbial community structure and functional activities (Wang et al., 2023). However, how hydrological connectivity shapes nitrogen pollution sources and microbial community structure in a river-lake connected at the watershed scale remains poorly understood. Therefore, the main objectives of this study were: (1) to clarify the spatiotemporal variations of nitrogen pollution loads and sources in the river-lake connected system; (2) to elucidate the spatiotemporal variations of the microbial community in the connected system; and (3) to establish the relationship between river-lake connectivity, nitrogen pollution, and microbial community dynamics. The objective of this study was to comprehensively understand the impacts of hydrological connectivity on nitrogen pollution sources and microbial community structure in a river-lake connected system.

## 2 Materials and methods

### 2.1 Study area and samples collection

**Figure 1:**
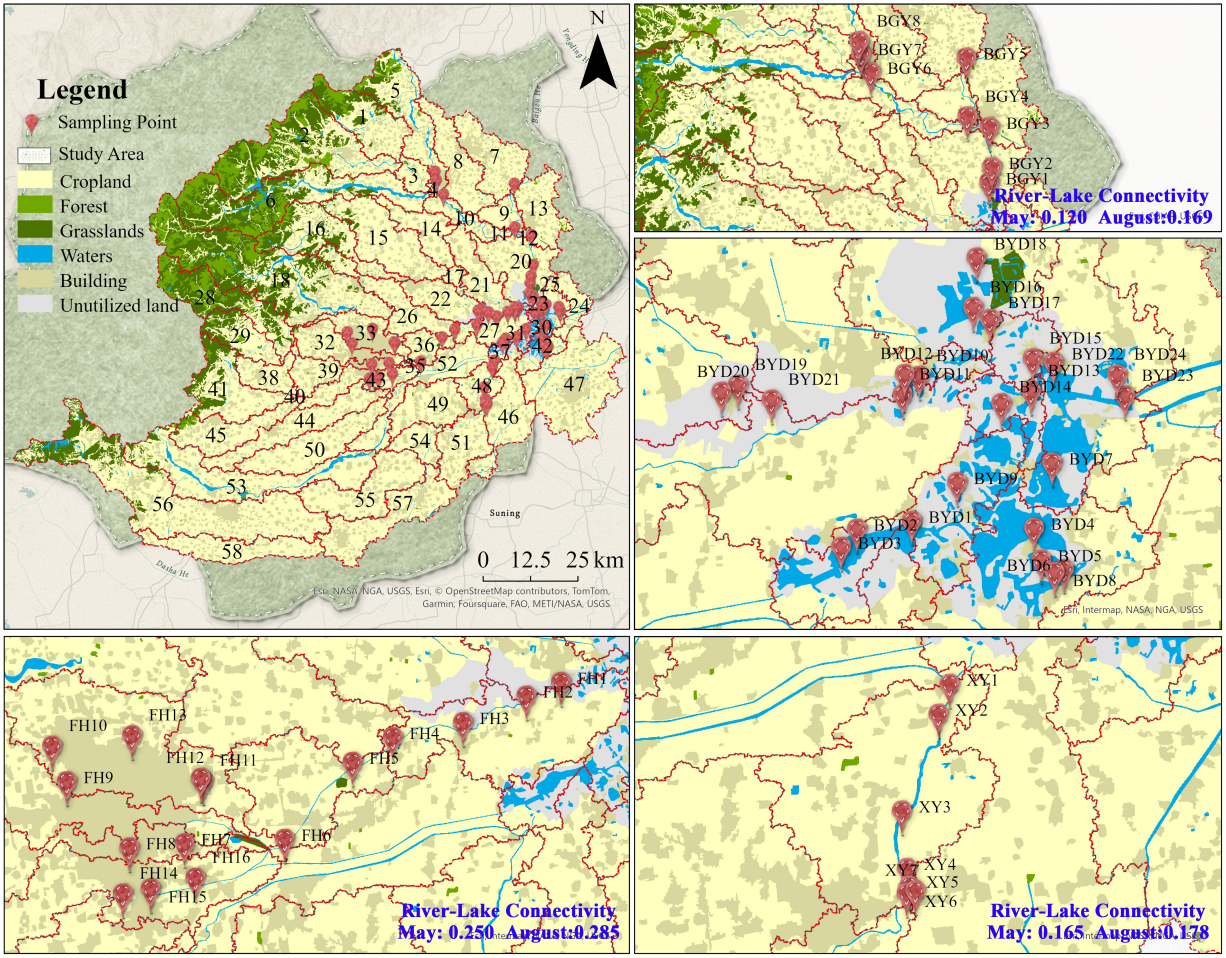
The sampling sites and land use types in the Baiyangdian watershed.

The study was conducted in Baiyangdian Lake (BYD) and its three main inflowing rivers: Fu River (FH), Baigouyin River (BGY), and Xiaoyi River (XY). BYD is a significant freshwater resource on the North China Plain, playing a crucial role in the local wetland ecosystem and the nitrogen cycling process Supplementary Text S1. This research selected rivers that represent various nitrogen pollution patterns in BYD and its inflowing rivers. The FH exhibited clear characteristics of PS pollution, the XY was significantly influenced by NPS pollution, and the BGY demonstrated features of mixed pollution from both PS and NPS. Sampling was carried out in May and August 2023, which represent the transition between the dry and wet seasons and the peak of the wet season, respectively. Totally 110 sampling sites were collected at two seasons, thereinto, 16 sites in FH, 7 sites in XY, 8 sites in BGY, and 24 sites in BYD (Figure 1). At each sampling site, both water and sediment samples were simultaneously collected. Water samples (collected at a depth of 10–30 cm) were gathered in triplicate using pre-cleaned polyethylene bottles, while sediment samples were taken from the top 0–5 cm of the surface sediment in triplicate using a grab sampler (XDB0201, Pusen, China). Both the water and sediment samples were homogenized, placed in pre-sterilized bags, and stored at −80°C for subsequent analysis. After sample collection, bacterial DNA was extracted from the sediment samples, and physicochemical analyses were performed on both the water and sediment samples.

### 2.2 Measurement of physicochemical parameters

In this study, a total of 10 environmental parameters were monitored. The concentrations of nitrate (${\text{NO}}_{3}^{-}$-N), nitrite (${\text{NO}}_{2}^{-}$-N), ammonium nitrogen (${\text{NH}}_{4}^{+}$-N), total phosphorus (TP) and total nitrogen (TN) in both water and sediment were quantified using the methods listed in the Supplementary Text S2.

### 2.3 DNA extraction and sequencing analysis

DNA was extracted from the homogenized sediment samples using a genomic DNA extraction kit specifically designed for sediments (Solarbio, Beijing, China). The quality of the extracted DNA was evaluated via 1% agarose gel electrophoresis, after which it was stored at −20°C for long-term preservation. PCR amplification and high-throughput sequencing were performed by Majorbio Bio-Pharm Technology Co., Ltd. (Shanghai, China). The amplification targeted the V3-V4 regions of the 16S rRNA gene, using the primers 806R (5′-GGACTACHVGGGTWTCTAAT-3′) and 338F (5′-ACTCCTACGGGAGGCAGCAG-3′) (detailed in Supplementary Text S3) (Curry et al., 2022). The sample sequence data have been deposited in the Sequence Read Archive (http://www.ncbi.nlm.nih.gov/sra/) for public access (Bioproject number: PRJNA1212803).

### 2.4 Isotope source analysis model (SIAR)

In this study, the SIAR isotope source analysis model was employed to estimate the contribution of each nitrogen pollution source. The SIAR model is represented by the following equation:


(1) $$\begin{array}{c} X_{ij}=\frac{\overset{K}{\underset{k=1}{\sum }}p_{k}q_{jk}\left(S_{jk}+C_{jk}\right)}{\overset{K}{\underset{k=1}{\sum }}p_{k}q_{jk}}+\varepsilon_{ij}\\ S_{jk}\sim N\left(\mu_{jk},\omega_{jk}^{2}\right)\\ C_{jk}\sim N\left(\lambda_{jk},\tau_{jk}^{2}\right)\\ \varepsilon_{jk}\sim N\left(0,\sigma_{j}^{2}\right) \end{array}$$

*X*_*jk*_ is the value of the jth isotope in the ith sample (i = 1,2,3,…, N; j = 1,2,3,…,J); *S*_*jk*_ is the value of the jth isotope in the kth source (k = 1,2,3,…,K); is the mean value; $\omega_{jk}^{2}$ is the variance of the normal distribution; C_*jk*_ is the fractionation factor of the jth isotope on the kth source; λ_*jk*_ is the average value; $\tau_{jk}^{2}$ is the variance of the normal distribution; *p*_*k*_ is the contribution of the kth source, which is calculated by the model; *q*_*jk*_ is the concentration of isotope j in the kth seed source; is the residual, which represents the remaining unquantified variation among the mixtures, with a mean of 0; $\sigma_{j}^{2}$ is the variance of the normal distribution (Stock et al., 2018).

### 2.5 SWAT model establishment

The SWAT model integrates land use data, soil characteristics, digital elevation model (DEM) data, meteorological information, daily stream flow records, and monthly monitoring data for TN, ${\text{NH}}_{4}^{+}$-N, ${\text{NO}}_{2}^{-}$-N and ${\text{NO}}_{3}^{-}$-N (as outlined in Supplementary Figure S1 and Supplementary Text S4). The sources of water pollution include both PS and NPS. PS can be divided into industrial zones, municipal wastewater treatment plants, rural sewage treatment facilities, and industrial wastewater discharges (Supplementary Text S5 and Supplementary Table S1). NPSs include crop cultivation (Supplementary Table S2), scattered small-scale animal feeding operations, and untreated rural sewage (Supplementary Text S5).

The SWAT-CUP software (Calibration and Uncertainty Programs) was used for model calibration and validation (Supplementary Text S6) (Abbaspour et al., 2015). In this process, the selected parameters were calibrated through 80,000 simulation iterations, as detailed in Supplementary Table S3. The model successfully simulated monthly discharge and nitrogen loads during both the calibration and validation phases, with *R*^2^ values exceeding 0.77 and Nash-Sutcliffe Efficiency (NSE) values >0.72. Further details on the nitrogen source attribution method can be found in the Supplementary Text S7.

### 2.6 Statistical analyses

A variety of statistical techniques were applied to analyze the data. Mantel tests, Canonical Correspondence Analysis (CCA), Principal Coordinates Analysis (PCoA), and Redundancy Analysis (RDA) were conducted using the vegan package in R (version 4.1.2). SPSS (version 26.0) was utilized for Wilcoxon rank-sum tests, Kruskal-Wallis tests, one-way ANOVA, and Spearman's correlation analysis. Additionally, Random Forest analysis was performed using the randomForest package in R (version 4.1.2). The phenotype and metabolic functions of the microbial community were predicted with the BugBase and Faprotax databases, along with annotations of prokaryotic taxon functions. BugBase was selected for its ability to predict the ecological characteristics of microbial communities, while Faprotax was chosen for its specific prediction of microbial metabolic functions such as nitrogen cycling. Linear Discriminant Analysis Effect Size (LEfSe) and bipartite association network analysis were also carried out. The co-occurrence network was visualized using Gephi software, and Structural Equation Modeling (SEM) was performed using the piecewiseSEM package in R.

## 3 Results

### 3.1 Temporal and spatial variations in total nitrogen and its forms

Temporally, the mean concentrations of TN, ${\text{NO}}_{3}^{-}$-N, ${\text{NO}}_{2}^{-}$-N, and ${\text{NH}}_{4}^{+}$-N in the connected system during the dry season were 1.94 ± 1.22 mg/l, 1.42 ± 1.91 mg/l, 0.11 ± 0.09 mg/l, and 0.23 ± 0.44 mg/l, respectively (Supplementary Figure S2). In the wet season, these concentrations increased to 2.62 ± 1.11 mg/l, 1.24 ± 1.67 mg/l, 0.12 ± 0.11 mg/l, and 0.29 ± 0.36 mg/l, respectively. During the wet season, TN, ${\text{NO}}_{2}^{-}$-N, and ${\text{NH}}_{4}^{+}$-N concentrations increased, whereas ${\text{NO}}_{3}^{-}$-N concentrations decreased slightly, with ${\text{NO}}_{2}^{-}$-N exhibited the most significant temporal variation (ANOVA, *p* < 0.05).

**Figure 2:**
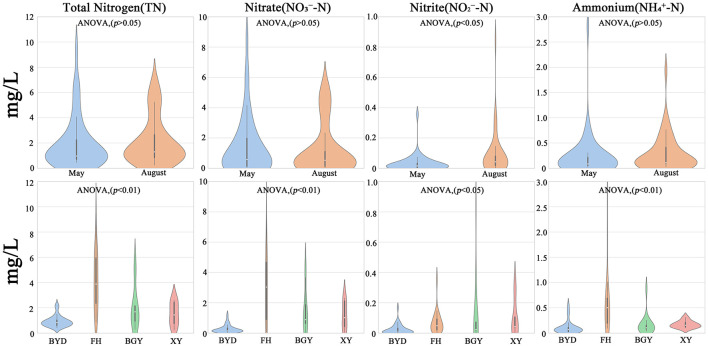
Seasonal and spatial variations of nitrogen components in samples.

Spatially, significant differences in nitrogen concentrations were observed among the regions (ANOVA, *p* < 0.05). The FH region consistently exhibited the highest nitrogen concentrations in both seasons. In contrast, the BGY and XY regions showed moderate nitrogen levels, while the BYD region, serving as the inflow lake, displayed the lowest TN, ${\text{NO}}_{3}^{-}$-N, ${\text{NO}}_{2}^{-}$-N, and ${\text{NH}}_{4}^{+}$-N concentrations. This indicates that the BYD lake system plays a crucial role in nitrogen dilution and retention, in contrast to the pollution characteristics of the riverine regions (Figure 2).

**Figure 3:**
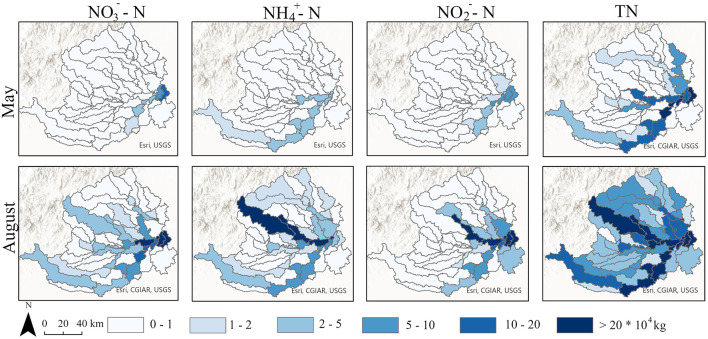
Spatial distribution of different nitrogen species in May and August simulated by the SWAT Model.

The SWAT model results revealed significant temporal variations in nitrogen loads between the two seasons (ANOVA, *p* < 0.05). During wet season, TN loads increased substantially across all regions. Specifically, in FH, the TN load rose from 986,942 to 6,389,722 kg; in BGY, it increased from 1,080,121 to 4,261,240 kg; and in XY, it grew from 210,681 to 1,426,230 kg. Similar temporal increases were observed for ${\text{NO}}_{3}^{-}$-N and ${\text{NH}}_{4}^{+}$-N. In FH, ${\text{NO}}_{3}^{-}$-N and ${\text{NH}}_{4}^{+}$-N loads increased from 34,499 to 788,545 kg and from 173,722 to 1,219,934 kg, respectively. In BGY, these loads increased from 9,765 to 372,540 kg and from 11,526 to 87,421 kg, respectively, and in XY, they increased from 2,341 to 105,740 kg and from 5,124 to 45,231 kg (Figure 3). Overall, nitrogen loads showed a significant increase during the wet season, with the most notable changes observed in FH.

The SWAT model accuracy was evaluated by comparing the observed versus predicted nitrogen loads, and a satisfactory fit was achieved (*R*^2^ > 0.85). The model results revealed significant spatial variations in nitrogen loads among the three sub-basins (ANOVA, *p* < 0.01). The spatial distribution consistently followed the tend: FH > BGY > XY. In dry season, the TN load in FH was 1,080,121 kg, which was higher than that in BGY (986,942 kg) and XY (210,681 kg). This pattern became even more pronounced in the wet season, when the TN load in FH increased to 6,389,722 kg, compared to 4,261,240 kg in BGY and 1,426,230 kg in XY. ${\text{NO}}_{3}^{-}$-N and ${\text{NH}}_{4}^{+}$-N loads displayed similar spatial trends, with FH showing the highest loads in both seasons, followed by BGY and XY. Additionally, ${\text{NO}}_{3}^{-}$-N loads exhibited clear spatial variations, with the loads in FH being the highest, particularly during the wet season, which served as the primary nitrogen output source. These findings demonstrate significant temporal and spatial variations in nitrogen loads.

### 3.2 Temporal and spatial variations in nitrate nitrogen sources

**Figure 4:**
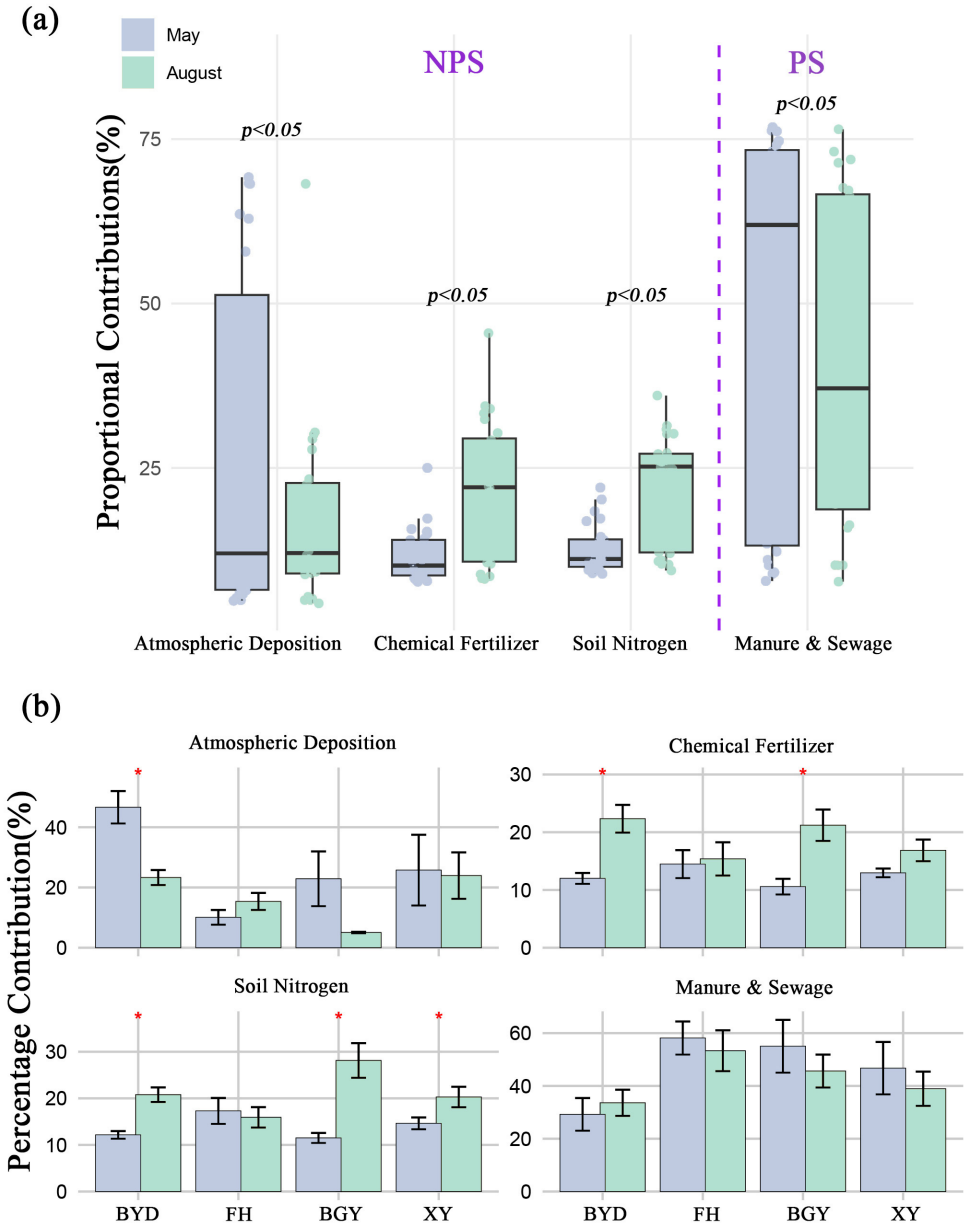
**(A)** Seasonal variations in proportional contributions of nitrogen sources, highlighting non-point source (NPS), and point source (PS) contributions during May and August; **(B)** Spatial distribution of proportional nitrogen source contributions (Atmospheric Deposition, Chemical Fertilizer, Soil Nitrogen, and Manure & Sewage) across different regions (BYD, FH, BGY, and XY) in May and August. ^*^p < 0.05.

The δ^15^N-${\text{NO}}_{3}^{-}$ values ranged from −9.6 to 44.6‰ (mean: 8.94 ± 5.00‰) during the dry season and from −6.6 to 21.7‰ (mean: 7.54 ± 4.00‰) during the wet season. Significant spatial differences were observed, with the mean δ^15^N-${\text{NO}}_{3}^{-}$ values in river systems being higher (dry season: 10.02 ± 4.75‰; wet season: 8.35 ± 4.20‰) than those in lake systems (dry season: 7.12 ± 5.25‰; wet season: 6.10 ± 4.50‰). Additionally, δ^18^O-${\text{NO}}_{3}^{-}$ values during the dry season (20.77 ± 4.14‰) were significantly higher than those during the wet season (−1.42 ± 3.50‰). δ^18^O-${\text{NO}}_{3}^{-}$ values were also higher in lake systems (dry season: 24.35 ± 5.20‰; wet season: −1.20 ± 3.40‰) compared to river systems, where the variation was less marked (dry season: 15.40 ± 5.00‰; wet season: −1.60 ± 3.20‰) (Supplementary Table S5). Using the MixSIAR model, the relative contributions of different nitrogen sources in rivers and lakes were estimated based on δ^15^N-${\text{NO}}_{3}^{-}$ and δ^18^O-${\text{NO}}_{3}^{-}$ values. The results identified four potential nitrogen sources in both seasons (Figures 4A, B). For further analysis, the sources were classified into two categories: NPS, which comprises atmospheric deposition (AP), chemical fertilizer (CF), and soil nitrogen (SN); and NP, which includes manure and sewage (M&S) (Gao et al., 2021; Ji et al., 2017; Kang et al., 2021; Yu et al., 2020). NPS showed an increasing trend from the dry to the wet season, whereas PS contributions decreased during the wet season in the connected system (Supplementary Figure S3). In terms of temporal variation, significant differences were observed in the contributions of NPS and PS between the dry and wet seasons (ANOVA, *p* < 0.01). Spatially, the contributions of NPS and PS also varied significantly between river and lake systems (ANOVA, *p* < 0.01).

### 3.3 Temporal and spatial variations in microbial community composition

The α-diversity analysis of microbial communities revealed significant temporal variations in species richness (Chao1), diversity (Shannon), and evenness (Simpson) between the dry and wet seasons (Supplementary Figure S5). Specifically, the Chao1 index ranged from 2,551 to 4,653 in the dry season from 2,941 to 5,763 in the wet season, with a notable difference observed in the BGY region (ANOVA, *p* < 0.05). The Shannon index ranged from 4.18 to 6.92 in the dry season and from 3.58 to 7.08 in the wet season, reflecting shifts in microbial diversity across the two seasons. The Simpson index, however, exhibited an opposite trend, with values ranging from 0.0035 to 0.1247 in the dry season and from 0.0034 to 0.1558 in the wet season, indicating significant temporal differences in microbial evenness among three rivers (ANOVA, *p* < 0.05).

**Figure 5:**
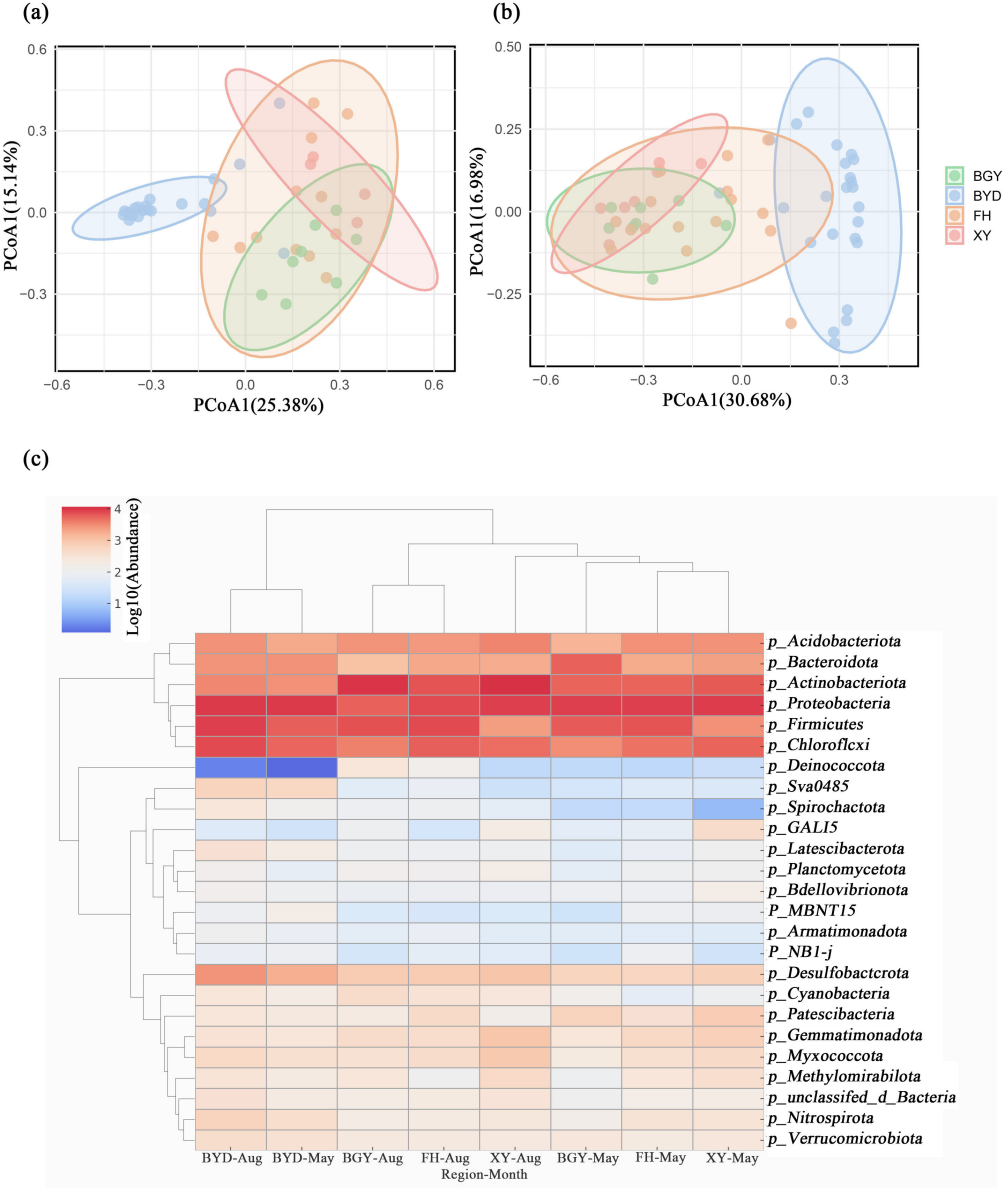
Characterization of microbial communities in BGY, BYD, FH, and XY in May and August. **(A, B)** Principal Coordinates Analysis (PCoA) of microbial communities in May and August; **(C)** Top 25 Community composition at the phylum level in May and August.

To further examine the changes in microbial community structure, taxonomic analysis was performed across the FH, BGY, XY, and BYD regions during both seasons (Supplementary Figure S4). The number of detected phyla increased from 64 in the dry season to 69 in the wet season, suggesting a greater community richness in the wet season. Cluster analysis further confirmed these changes, showing difference in microbial community structures between the rivers and the lake (ANOVA, *p* < 0.05). In both seasons, *Proteobacteria, Firmicutes*, and *Actinobacteriota* were the dominant phyla at all sampled sites During the dry season, *Proteobacteria* was the most abundant (26.73%), followed by *Firmicutes* (17.28%) and *Actinobacteriota* (12.88%). However, in the wet season, the relative abundance of *Proteobacteria* decreased to 20.73%, while those of *Firmicutes* and *Actinobacteriota* increased to 18.55 and 16.13%, respectively (Supplementary Figure S6). The ANOSIM analysis indicated significant spatial differences in microbial community composition in both seasons (*p* < 0.01, 999 permutations). Notably, the highest microbial abundance was observed in the BYD region, followed by FH, BGY, and XY. PCoA analysis also revealed clear separation of microbial community structures among these regions in both seasons. In the dry season, PCoA1 and PCoA2 accounted for 25.38 and 15.14% of the total variation, respectively. In the wet season, these axes explained 30.68 and 16.98% of the variation, with BYD showing a more concentrated distribution along the PCoA1 axis compared to other regions (Figures 5A, B).

Spatial variations in the relative abundance of dominant phyla were observed across regions (Figure 5C). During the dry season, *Firmicutes* (17.28%) was most abundant in FH, where it plays a crucial role in organic matter decomposition. In contrast, *Proteobacteria* dominated the microbial communities in BGY (25.33%), XY (26.63%), and BYD (26.13%). In the wet season, the abundance of *Proteobacteria* decreased to 20.73%, while *Actinobacteriota* (16.13%) became the most abundant phylum in BYD. This shift in microbial community composition reflects temporal changes in microbial diversity across regions. Additionally, some microbial communities with increased abundance were identified in FH, BGY, and XY. For example, the abundance of *Nitrospirota*, a phylum associated with nitrification and nitrogen cycling, increased from 0.097% in the dry season to 1.01% in the wet season. This increase may be linked to dynamic changes in nitrogen cycling processes during the wet season and microbial niche competition under eutrophic conditions. The increased abundance of *Nitrospirota* suggests that nitrification processes, which are crucial for nitrogen cycling, are more active in the wet season.

Mantel test revealed that the ${\text{NO}}_{3}^{-}$-N concentration in water (*R* = 0.587, *p* = 0.0562) was the most significant factor associated with microbial community composition. CCA further indicated that the TN concentration in sediment, which had the highest *R*^2^ value, was the most influential factor for microbial community composition (*R*^2^ = 0.459, *p* = 0.0291). Additionally, ammonia nitrogen and nitrite exhibited strong correlations with microbial populations (Supplementary Figure S7). These findings support the hypothesis that nitrogen concentration is a key factor influencing microbial community structure in the Baiyangdian watershed.

### 3.4 Association between contaminant transport mode and microbial characteristics

**Figure 6:**
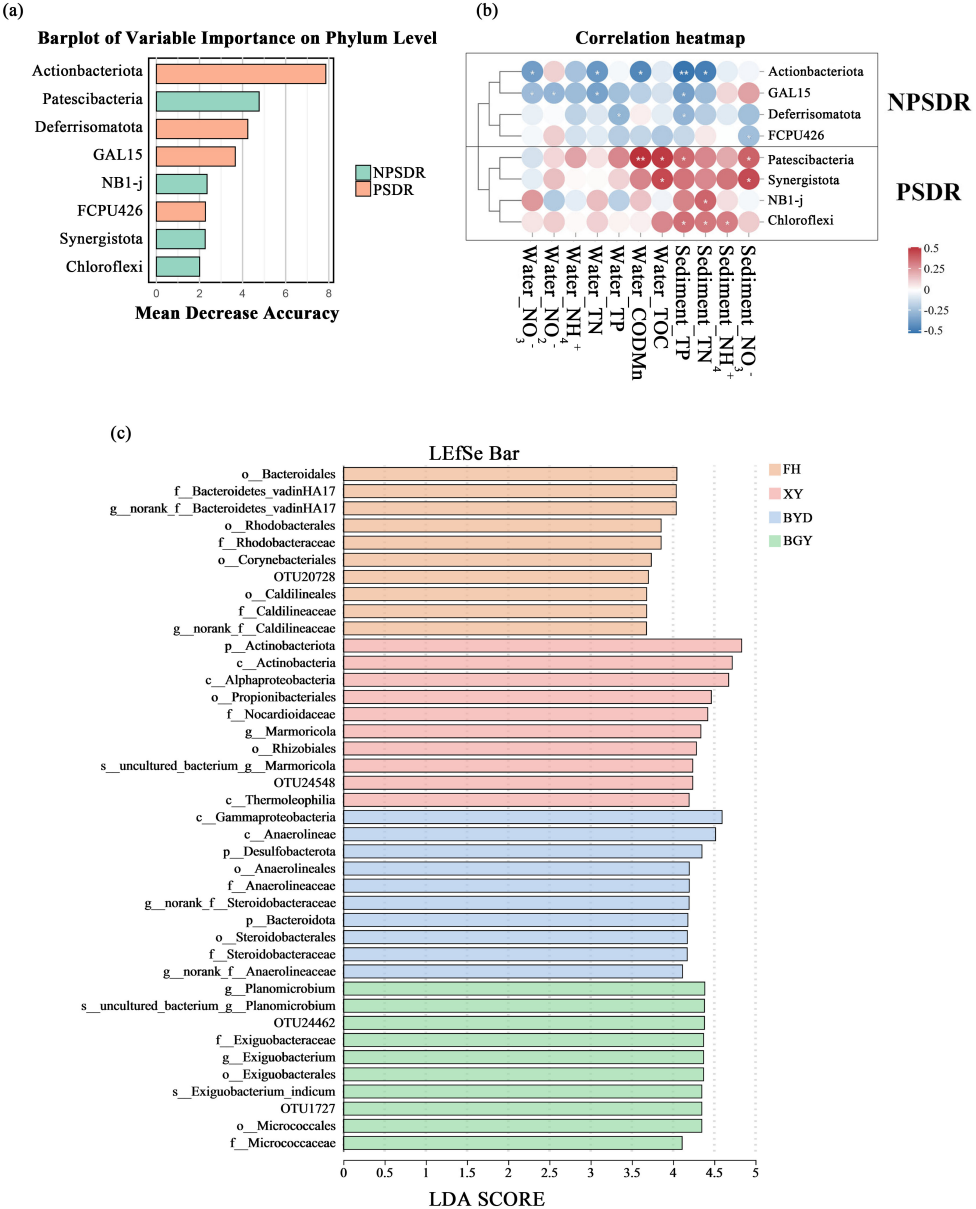
**(A)** Barplot of variable importance on phylum level, showing the most influential phyla distinguishing between NPSDR and PSDR groups; **(B)** Correlation heatmap between selected phyla and environmental factors, comparing NPSDR and PSDR groups; **(C)** LEfSe analysis identifying significant microbial taxa across different river sites (BGY, BYD, FH, and XY).

Based on previous analysis, the rivers were classified into three categories: PS-dominated river (PSDR), NPS-dominated river (NPSDR), and balanced pollution river (BPR). The FH is classified as a typical PSDR, primarily influenced by urban sewage and industrial discharges. In contrast, the BGY exhibits characteristics of an NPSDR, significantly impacted by agricultural runoff and soil nitrogen sources. The XY River was identified as a BPR, reflecting the combined effects of multiple pollution sources. Based on the PCoA results (Figures 5A, B), the samples from BGY, BYD, FH, and XY were clustered into four distinct groups. Furthermore, the LEfSe analysis (Figure 6C) revealed specific microbial taxa as significant biomarkers distinguishing these rivers.

The microbial communities in the NPSDR exhibited significantly higher species richness and community diversity (*p* < 0.05; Wilcoxon rank-sum test), indicating increased within-habitat diversity in the NPSDR. Compared with the PSDR and BPR, the NPSDR also showed a significantly greater number of microbial species (Supplementary Figure S8). The findings suggested that the microbial communities in the PSDR experienced a reduction in both population size and diversity, potentially favoring certain functional groups and leading to biotic homogenization.

In the random forests analysis, the eight most important features were used as biomarkers to distinguish between PSDR and NPSDR (Figure 6A), with the abundances of these biomarkers detailed in Supplementary Table S4. Four biomarkers, Patescibacteria, Synergistota, NBI-j, and Chloroflexi, were more prevalent in PSDR than in NPSDR, whereas the remaining biomarkers, Actinobacteriota, GAL15, Deferisomatota, and FCPU426, were more prevalent in NPSDR than in PSDR (Supplementary Figure S9). The correlation analysis between the relative abundance of these selected biomarkers and the measured physicochemical properties of the rivers revealed two distinct clusters (Figure 6B). The four biomarkers enriched in the PSDR showed positive correlations with most environmental factors (e.g., water nitrate, water nitrite, and total nitrogen in water), whereas the, four bacterial families from the NPSDR exhibited negative correlations with most environmental factors (e.g., sediment nitrate, sediment ammonium). Consequently, rivers with different nitrogen contamination transport modes exhibited variability in sediment microenvironments and differences in microbial communities.

### 3.5 Influence of river-lake connectivity on nitrogen migration and microbial distribution

The river-lake connectivity index results revealed significant spatial differences among rivers in both seasons. The average connectivity index for FH was 0.250 in the dry season and 0.285 in the wet season, values that were significantly higher than those for BGY and XY (dry season: BGY = 0.120, XY = 0.165; wet season: BGY = 0.169, XY = 0.178).

**Figure 7:**
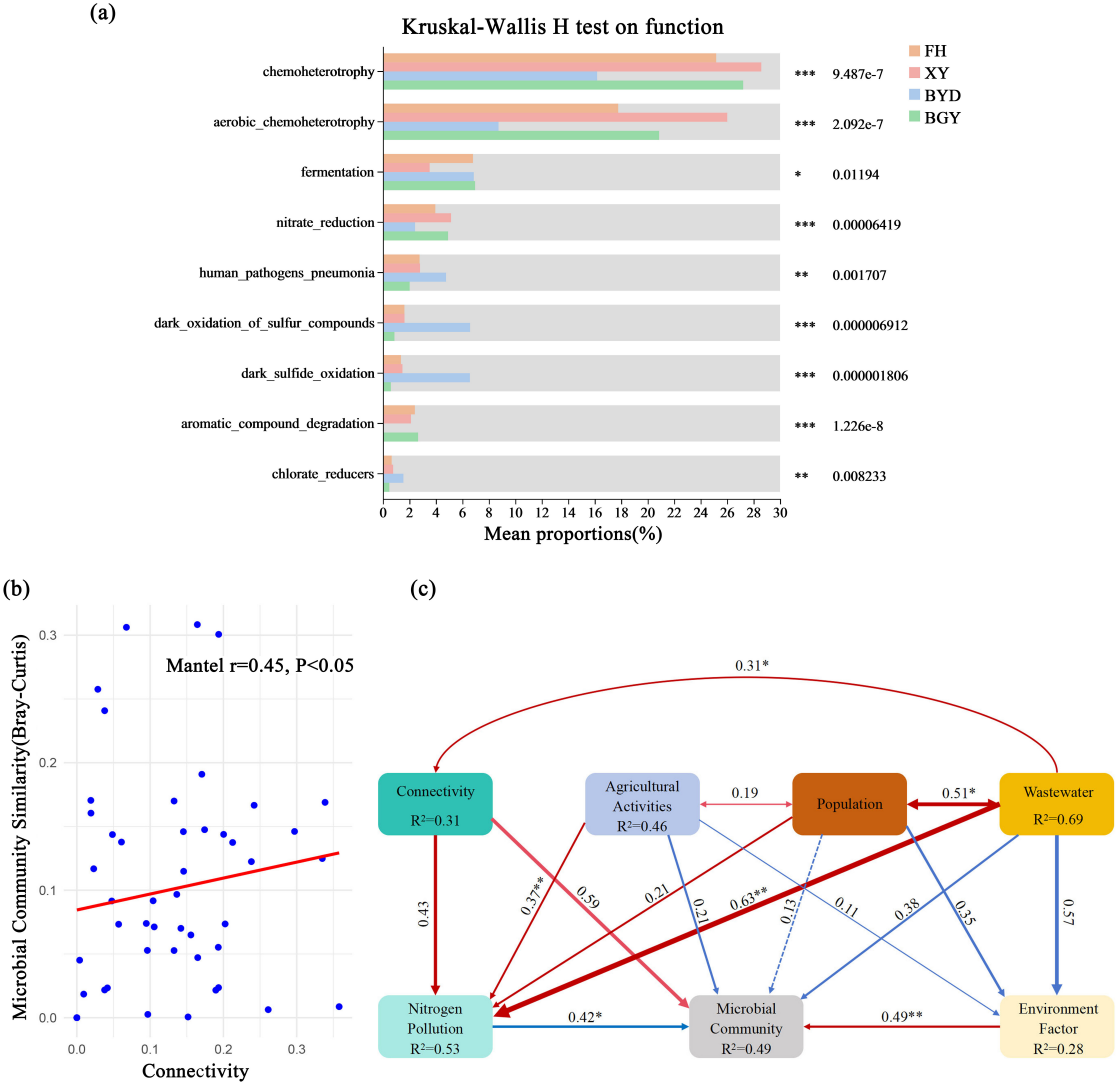
**(A)** Comparison of metabolic functions in the BGY, BYD, FH, and XY using FAPROTAX; **(B)** Relationship between connectivity and microbial community similarity. **(C)** Structural equation model of factors influencing nitrogen pollution and microbial community. ^*^p < 0.05, ^**^p < 0.01, ^***^p < 0.001.

The relationships between the river-lake connectivity index, TN, ${\text{NO}}_{3}^{-}$-N, ${\text{NH}}_{4}^{+}$-N concentrations, and microbial community α-diversity (Chao1 and Shannon indices) were examined. The results revealed significant positive correlations between the connectivity index and TN (*r* = 0.76, *p* < 0.01), ${\text{NO}}_{3}^{-}$-N (*r* = 0.79, *p* < 0.01), and ${\text{NH}}_{4}^{+}$-N (*r* = 0.75, *p* < 0.01) concentrations. In contrast, significant negative correlations were observed between the connectivity index and microbial community alpha diversity (Chao1: *r* = −0.74, *p* < 0.01; Shannon: *r* = −0.72, *p* < 0.01) (Supplementary Figure S10). These findings suggest that higher connectivity can enhance nitrogen migration and accumulation in water bodies, potentially affecting community diversity. Further Mantel tests indicated a significant positive correlation between connectivity and microbial community similarity (*r* = 0.45, *p* < 0.05) (Figure 7B). This finding suggests that connectivity plays a crucial role in shaping the spatial distribution of microbial communities, with higher connectivity associated with increased community composition similarity. Enhanced connectivity promotes the spread of specific functional microbes in water bodies, potentially reducing local species diversity (i.e., α-diversity). Functional diversity analysis further revealed differences in microbial functional characteristics among rivers. In high-connectivity and high-nitrogen rivers (e.g., FH), microbial communities were enriched with functions such as chemoheterotrophy, aerobic chemoheterotrophy, and nitrate reduction (Figure 7A), which are essential for the nitrogen cycle. This result suggests that river-lake connectivity can promote the dispersal of nitrogen-metabolizing microbes, thereby influencing nitrogen transformation and ecosystem function.

SEM was employed to further explore the relationships among connectivity, agricultural activities, population density, wastewater discharge, environmental factors, and microbial communities. The SEM results (Figure 7C) revealed significant direct and indirect interactions among these factors, demonstrating how connectivity and nitrogen pollution influence microbial distribution patterns. The model indicated that connectivity had a direct positive effect on nitrogen pollution (path coefficient = 0.43, *p* < 0.05), which can explaining 53% of the variation in nitrogen pollution. These results suggest that increased connectivity enhances nitrogen transport, leading to elevated nitrogen concentrations. Furthermore, nitrogen pollution directly influenced microbial communities (path coefficient = 0.42, *p* < 0.05), explaining 49% of the variance in microbial community structure, which indicates that nitrogen pollution can alter microbial composition by promoting the growth of nitrogen-metabolizing microbes. Agricultural activities (*R*^2^ = 0.46) and population density (*R*^2^ = 0.51) were also identified as significant drivers of nitrogen pollution, indirectly affecting microbial communities by increasing nitrogen load and wastewater discharge. In addition, the SEM results showed that wastewater discharge (*R*^2^ = 0.69), which was significantly influenced by population density (path coefficient = 0.51, *p* < 0.05), was one of the primary sources of nitrogen pollution. Moreover, environmental factors (*R*^2^ = 0.28) exhibited indirect effects on microbial communities by modulating nitrogen pollution pathways. These findings indicate that in river-lake systems, microbial diversity and composition in rivers with high nitrogen pollution and connectivity are predominantly influenced by connectivity and nitrogen pollution.

## 4 Discussion

### 4.1 Influence of nitrogen pollution sources and hydrological connectivity on spatiotemporal variations in nitrogen concentrations

This study revealed marked seasonal and spatial variations in nitrogen concentrations in the Baiyangdian watershed. During the wet season, nitrogen concentrations increased across the watershed, particularly in regions with high hydrological connectivity (e.g., FH). This increase was driven by intensified precipitation and surface runoff, which not only enhanced nitrogen transport but also altered the distribution of pollution sources. The elevated hydrological connectivity facilitated the transport of NPS pollutants, leading to a significant rise in nitrogen loads. In the wet season, compared to the dry season, concentrations of TN, ${\text{NH}}_{4}^{+}$-N, and ${\text{NO}}_{3}^{-}$-N exhibited substantial increases. These results are consistent with findings from other river basins, where increases in precipitation and runoff have been shown to mobilize and intensify nitrogen influx from both agricultural and urban sources into water bodies (Levy-Booth et al., 2014; Zhang T. et al., 2022). This underscores the critical role of hydrological conditions in governing the spatiotemporal dynamics of nitrogen pollution (Yin et al., 2018; Zhang X. et al., 2020).

Spatially, BYD exhibited relatively lower nitrogen concentrations during the wet season, indicating that the lake system plays an important role in buffering nitrogen pollution through dilution and biogeochemical transformation. This effect may be attributed to a combination of sedimentation, denitrification, and nutrient exchange processes between water and sediments (Li et al., 2020). In contrast, FH and BGY exhibited higher nitrogen concentrations, mainly influenced by agricultural runoff, urban wastewater, and industrial discharges. These spatial differences highlight the diverse nitrogen pollution sources across the watershed, with hydrological connectivity playing a crucial role in the migration and accumulation of pollutants. Regions with stronger connectivity and concentrated water flow, such as FH, not only experience increased nitrogen transport but also demonstrate higher retention of nitrogen loads, exacerbating local pollution (Masoner et al., 2023).

The SWAT model results further validate the significant role of hydrological connectivity in nitrogen load transmission. High-connectivity regions, particularly FH, experienced substantial nitrogen accumulation, indicating that increased water flow not only expedites nitrogen dispersal but also amplifies nitrogen deposition in specific areas. This finding is consistent with studies conducted in other watersheds, which have emphasized the importance of hydrological connectivity in regulating the spatial distribution of nitrogen pollution (Lei et al., 2024; Wen Y. et al., 2024). However, it is important to acknowledge that while the SWAT model effectively estimates nitrogen transport patterns and hydrological connectivity, it does not explicitly simulate hydrodynamic variables such as flow velocity and turbulence intensity.

### 4.2 Influence of nitrogen sources on microbial community structure

The significant seasonal differences in nitrogen source contributions, with NPS playing a more dominant role in the wet season, while PS were more prevalent in the dry season. These seasonal variations are likely linked to increased surface runoff during the wet season, which transports more nitrogen from fertilizers and soil into the water system (Cao et al., 2024). This finding underscores the influence of hydrological conditions on the seasonal dynamics of nitrogen sources, with precipitation intensity playing a key role in the contribution of NPS pollution (Li et al., 2019; Soana et al., 2024).

Microbial community composition was closely associated with seasonal changes in nitrogen sources. The increased abundance of nitrifying bacteria (e.g., *Nitrospirota*) in the wet season indicates the strong influence of seasonal nitrogen inputs on microbial nitrogen metabolism (Zhang Y. et al., 2022). These findings suggest that elevated nitrogen concentrations in the wet season directly stimulate the proliferation of nitrogen-metabolizing microorganisms, thereby enhancing nitrogen transformation processes (Ren et al., 2024). In contrast, in low-nitrogen regions such as BYD, the microbial community was predominantly composed of nitrogen-fixing bacteria, characterized by higher abundances of Actinobacteria and Cyanobacteria. These nitrogen-fixing microbes contribute to the biological fixation of atmospheric nitrogen, converting it into a usable nitrogen source and maintaining nitrogen cycling in low-nitrogen environments (Zhao et al., 2022). This shift in microbial community composition and functionality underscores the significant impact of nitrogen sources on microbial community structure and ecosystem function (Zhu et al., 2023; Sun et al., 2024).

### 4.3 Influence of river-lake connectivity on nitrogen migration and microbial distribution

The results of this study indicate that river-lake connectivity plays a critical role in the migration of nitrogen and the distribution of microbial communities in the Baiyangdian watershed. These findings are consistent with previous studies demonstrating that river-lake connectivity significantly influences the transport of nutrients and pollutants, thereby affecting both water quality and ecosystem functioning (Zhang Z. et al., 2020; Wen Y. et al., 2024).

The analysis indicates that microbial community α-diversity is significantly negatively correlated with the river-lake connectivity index. This phenomenon may be attributed to several factors, including hydrodynamic disturbances, changes in nitrogen sources, and alterations in water quality. Higher river-lake connectivity increases hydrodynamic turbulence and physical mixing, potentially disrupting native microbial habitats by accelerating water flow, and thereby disturbing the microbial community structure. In this process, microorganisms that are better adapted to dynamic hydrological conditions may dominate the community, resulting in homogenization and a subsequent reduction in diversity. Furthermore, enhanced river-lake connectivity can significantly alter the types of nitrogen sources in rivers and lakes. For example, in FH regions, urban wastewater discharge provides a substantial nitrogen source. Increased connectivity accelerates nitrogen migration and accumulation, promoting the growth of nitrogen-metabolizing microorganisms, such as nitrifiers and denitrifiers, which further alters the microbial community composition and decreases diversity. Additionally, higher river-lake connectivity may affect water quality indicators, such as dissolved oxygen, transparency, and pH, thereby further influencing the microbial habitat. For instance, a decrease in dissolved oxygen may favor the growth of anaerobic microorganisms while inhibiting the proliferation of aerobic microbes. These findings are consistent with previous studies, which suggest that river-lake connectivity plays a crucial role in the transport of nutrients and pollutants, thereby significantly shaping microbial community composition and function (Freimann et al., 2015; Pan et al., 2022).

Furthermore, SEM analysis revealed that river-lake connectivity had a significant indirect effect on microbial community structure through nitrogen concentrations. The model showed a positive path coefficient (0.43, *p* < 0.05) between connectivity and nitrogen pollution, with nitrogen pollution further influencing microbial diversity (0.42, *p* < 0.05). This finding suggests that enhanced river-lake connectivity accelerates the migration of nitrogen, which in turn alters microbial community composition, leading to changes in microbial diversity and ecosystem functions (Crevecoeur et al., 2023). These results emphasize the complex interplay among hydrological connectivity, nitrogen pollution, and microbial communities, highlighting the need for integrated management strategies that consider both physical and ecological connectivity.

## 5 Conclusion

In summary, this study reveals the relationship among nitrogen pollution sources, hydrological connectivity, and microbial community diversity. The results show significant seasonal variations in nitrogen sources, with NPS such as agricultural fertilizers and soil nitrogen contributing more in the wet season, whereas PS dominate in the dry season. Hydrological connectivity plays a key role in nitrogen migration and microbial community distribution, particularly in regions with high connectivity, such as FH. These areas exhibit higher nitrogen concentrations and reduced microbial diversity, indicating that hydrological connectivity accelerates the spread of pollutants and facilitates the proliferation of specific functional microbes, leading to microbial community homogenization. Microbial community analysis revealed that high-nitrogen regions, such as FH and BGY, were enriched with nitrogen-metabolizing bacteria, including *Proteobacteria* and *Firmicutes*, which contribute to nitrogen cycling and water self-purification. In contrast, in low-nitrogen environments such as BYD, the microbial community was predominantly composed of nitrogen-fixing bacteria, such as Actinobacteria and Cyanobacteria, which play a key role in maintaining nitrogen cycling through biological nitrogen fixation. Overall, the interactions among nitrogen pollution sources, hydrological connectivity, and microbial communities exhibit significantly spatial-temporal difference.

## Data Availability

The datasets presented in this study can be found in online repositories. The names of the repository/repositories and accession number(s) can be found below: https://www.ncbi.nlm.nih.gov/, PRJNA1A212803.

## References

[B1] Abbaspour K. C. Rouholahnejad E. Vaghefi S. Srinivasan R. Yang H. Kløve B. (2015). A continental-scale hydrology and water quality model for Europe: calibration and uncertainty of a high-resolution large-scale SWAT model. J. Hydrol. 524, 733–752. 10.1016/j.jhydrol.2015.03.027

[B2] Arshad Z. Shin K. H. Hur J. (2025). Utilization and applications of stable isotope analysis for wastewater treatment systems: a review. Environ. Res. 264:120347. 10.1016/j.envres.2024.120347 39528035

[B3] Cao W. Li Q. Xu H. Zhang Z. (2024). Vegetation dynamics regulate baseflow seasonal patterns of the chaohe watershed in north China. J. Hydrol. Reg. Stud. 53:101797. 10.1016/j.ejrh.2024.101797

[B4] Crevecoeur S. Edge T. A. Watson L. C. Watson S. B. Greer C. W. Ciborowski J. J.. (2023). Spatio-temporal connectivity of the aquatic microbiome associated with cyanobacterial blooms along a great lake riverine-lacustrine continuum. Front. Microbiol. 14:1073753. 10.3389/fmicb.2023.1073753 36846788 PMC9947797

[B5] Curry K. D. Wang Q. Nute M. G. Tyshaieva A. Reeves E. Soriano S.. (2022). Emu: species-level microbial community profiling of full-length 16S rRNA oxford nanopore sequencing data. Nat. Methods 19, 845–853. 10.1038/s41592-022-01520-4 35773532 PMC9939874

[B6] Forgrave R. Evenson G. R. Golden H. E. Christensen J. R. Lane C. R. Wu Q.. (2024). Wetland-mediated nitrate reductions attenuate downstream: insights from a modeling study. J. Environ. Manag. 370:122500. 10.1016/j.jenvman.2024.122500 39299124 PMC11568908

[B7] Freimann R. Bürgmann H. Findlay S. E. Robinson C. T. (2015). Hydrologic linkages drive spatial structuring of bacterial assemblages and functioning in alpine floodplains. Front. Microbiol. 6:1221. 10.3389/fmicb.2015.01221 26579113 PMC4630579

[B8] Gao J. Li Z. Chen Z. Zhou Y. Liu W. Wang L.. (2021). Deterioration of groundwater quality along an increasing intensive land use pattern in a small catchment. Agric. Water Manage. 253:106953. 10.1016/j.agwat.2021.106953

[B9] Guo J. Pan Y. Chen R. He S. Qi W. Yang H.. (2024). Tracing nitrogen and phosphorus pollution in urban runoff: insights from isotopic tracers and SWMM modeling. J. Clean. Prod. 472:143513. 10.1016/j.jclepro.2024.143513

[B10] Ji X. Xie R. Hao Y. Lu J. (2017). Quantitative identification of nitrate pollution sources and uncertainty analysis based on dual isotope approach in an agricultural watershed. Environ. Pollut. 229, 586–594. 10.1016/j.envpol.2017.06.100 28689147

[B11] Kang P. Li S. Wang F. Zhao H. Lv S. (2021). Use of multiple isotopes to evaluate nitrate dynamics in groundwater under the barrier effect of underground cutoff walls. Environ. Sci. Pollut. Res. 28, 7076–7089. 10.1007/s11356-020-10792-2 33025438

[B12] Lei M. Long Y. Li T. Ma Y. Zhang G. Peng B.. (2024). Nitrogen dynamic transport processes shaped by watershed hydrological functional connectivity. J. Hydrol. 645:132218. 10.1016/j.jhydrol.2024.132218

[B13] Levy-Booth D. J. Prescott C. E. Grayston S. J. (2014). Microbial functional genes involved in nitrogen fixation, nitrification and denitrification in forest ecosystems. Soil Biol. Biochem. 75, 11–25. 10.1016/j.soilbio.2014.03.021

[B14] Li B. Yang G. Wan R. (2020). Multidecadal water quality deterioration in the largest freshwater lake in China (Poyang lake): implications on eutrophication management. Environ. Pollut. 260:114033. 10.1016/j.envpol.2020.114033 32006887

[B15] Li C. Li S. L. Yue F. J. Liu J. Zhong J. Yan Z. F.. (2019). Identification of sources and transformations of nitrate in the yangtzer River using nitrate isotopes and bayesian model. Sci. Total Environ. 646, 801–810. 10.1016/j.scitotenv.2018.07.345 30064106

[B16] Masoner J. R. Kolpin D. W. Cozzarelli I. M. Bradley P. M. Arnall B. B. Forshay K. J.. (2023). Contaminant exposure and transport from three potential reuse waters within a single watershed. Environ. Sci. Technol. 57, 1353–1365. 10.1021/acs.est.2c07372 36626647 PMC9878729

[B17] Oulehle F. Cosby B. J. Austnes K. Evans C. D. Hruška J. Kopáček J.. (2015). Modelling inorganic nitrogen in runoff: seasonal dynamics at four European catchments as simulated by the MAGIC model. Sci. Total Environ. 536, 1019–1028. 10.1016/j.scitotenv.2015.05.047 26094110

[B18] Pan B. Liu X. Chen Q. Sun H. Zhao X. Huang Z. (2022). Hydrological connectivity promotes coalescence of bacterial communities in a floodplain. Front. Microbiol. 13:971437. 10.3389/fmicb.2022.971437 36212880 PMC9532515

[B19] Qian C. Wang Q. Gilfedder B. S. Frei S. Yu J. Kattel G. R.. (2025). Seasonal dynamics of groundwater discharge: unveiling the complex control over reservoir greenhouse gas emissions. Water Res. 269:122801. 10.1016/j.watres.2024.122801 39571523

[B20] Ren J. Zhao S. Xu L. Xie W. Meng H. He H.. (2024). Evidence of comammox bacteria playing a dominant role in lake Taihu sediments based on metagenomic analysis. Int. Biodeterior. Biodegrad. 193:105846. 10.1016/j.ibiod.2024.105846

[B21] Rudneva I. I. Omel'chenko S. O. (2021). Nitrosamines in aquatic ecosystems: sources, formation, toxicity, environmental risk (review) 1. Structure, properties, ways of entering and formation in waterbodies. Water Resour. 48, 92–101. 10.1134/S0097807821010255

[B22] Soana E. Gervasio M. P. Granata T. Colombo D. Castaldelli G. (2024). Climate change impacts on eutrophication in the Po River (Italy): temperature-mediated reduction in nitrogen export but no effect on phosphorus. J. Environ. Sci. 143, 148–163. 10.1016/j.jes.2023.07.008 38644013

[B23] Stock B. C. Jackson A. L. Ward E. J. Parnell A. C. Phillips D. L. Semmens B. X. (2018). Analyzing mixing systems using a new generation of Bayesian tracer mixing models. PeerJ 6:e5096. 10.7717/peerj.5096 29942712 PMC6015753

[B24] Sun Q. Li J. Zhou C. Jiang W. Lei K. (2024). The influence of river basin nitrogen pollution sources and their transport on microbial community structure. Front. Mar. Sci. 11:1459186. 10.3389/fmars.2024.1459186

[B25] Vrzel J. Vuković-Gačić B. Kolarević S. Gačić Z. Kračun-Kolarević M. Kostić J.. (2016). Determination of the sources of nitrate and the microbiological sources of pollution in the Sava River Basin. Sci. Total Environ. 573, 1460–1471. 10.1016/j.scitotenv.2016.07.213 27522292

[B26] Wang C. Wang X. Xu Y. J. Lv Q. Ji X. Jia S.. (2024). Multi-evidences investigation into spatiotemporal variety, sources tracing, and health risk assessment of surface water nitrogen contamination in China. Environ. Res. 262:119906. 10.1016/j.envres.2024.119906 39233034

[B27] Wang J. Li X. Li Y. Shi Y. Y. Xiao H. B. Wang L.. (2024). Transport pathways of nitrate in stormwater runoff inferred from high-frequency sampling and stable water isotopes. Environ. Sci. Technol. 58, 17026–17035. 10.1021/acs.est.4c02495 39152914

[B28] Wang M. Bodirsky B. L. Rijneveld R. Beier F. Bak M. P. Batool M.. (2024). A triple increase in global river basins with water scarcity due to future pollution. Nat. Commun. 15:880. 10.1038/s41467-024-44947-3 38321008 PMC10847517

[B29] Wang Y. Lin J. Wang F. Tian Q. Zheng Y. Chen N. (2023). Hydrological connectivity affects nitrogen migration and retention in the land-river continuum. J. Environ. Manag. 326:116816. 10.1016/j.jenvman.2022.116816 36417834

[B30] Wen M. Liu Y. Yang C. Dou Y. Zhu S. Tan G.. (2024). Effects of manure and nitrogen fertilization on soil microbial carbon fixation genes and associated communities in the Loess Plateau of China. Sci. Total Environ. 954:176. 10.1016/j.scitotenv.2024.176581 39368509

[B31] Wen Y. Lin J. S. Plaza F. Liang X. (2024). Roles of hydrology and transport processes in denitrification at watershed scale. *Water Resour*. Res. 60:e2023WR034971. 10.1029/2023WR034971

[B32] Xu P. Li G. Zheng Y. Fung J. C. Chen A. Zeng Z.. (2024). Fertilizer management for global ammonia emission reduction. Nature 626, 792–798. 10.1038/s41586-024-07020-z 38297125

[B33] Yin J. Gentine P. Zhou S. Sullivan S. C. Wang R. Zhang Y.. (2018). Large increase in global storm runoff extremes driven by climate and anthropogenic changes. Nat. Commun. 9:4389. 10.1038/s41467-018-06765-2 30348951 PMC6197252

[B34] Yu C. Huang X. Chen H. Godfray H. C. J. Wright J. S. Hall J. W.. (2019). Managing nitrogen to restore water quality in China. Nature 567, 516–520. 10.1038/s41586-019-1001-1 30818324

[B35] Yu L. Zheng T. Zheng X. Hao Y. Yuan R. (2020). Nitrate source apportionment in groundwater using bayesian isotope mixing model based on nitrogen isotope fractionation. Sci. Total Environ. 718:137242. 10.1016/j.scitotenv.2020.137242 32105927

[B36] Zhang S. Zhang L. Meng Q. Wang C. Ma J. Li H.. (2024). Evaluating agricultural non-point source pollution with high-resolution remote sensing technology and SWAT model: a case study in Ningxia Yellow River Irrigation District, China. Ecol. Indic. 166:112578. 10.1016/j.ecolind.2024.112578

[B37] Zhang T. Xu S. Yan R. Wang R. Gao Y. Kong M.. (2022). Similar geographic patterns but distinct assembly processes of abundant and rare bacterioplankton communities in river networks of the Taihu Basin. Water Res. 211:118057. 10.1016/j.watres.2022.118057 35066261

[B38] Zhang X. Ward B. B. Sigman D. M. (2020). Global nitrogen cycle: critical enzymes, organisms, and processes for nitrogen budgets and dynamics. Chem. Rev. 120, 5308–5351. 10.1021/acs.chemrev.9b00613 32530264

[B39] Zhang Y. Zhang Y. Wei L. Li M. Zhu W. Zhu L. (2022). Spatiotemporal correlations between water quality and microbial community of typical inflow river into Taihu Lake, China. Environ. Sci. Pollut. Res. 29, 63722–63734. 10.1007/s11356-022-19023-2 35460482

[B40] Zhang Z. Chen X. Cheng Q. Li S. Yue F. Peng T.. (2020). Coupled hydrological and biogeochemical modelling of nitrogen transport in the karst critical zone. Sci. Total Environ. 732:138902. 10.1016/j.scitotenv.2020.138902 32438160

[B41] Zhao F. Zhan X. Xu H. Zhu G. Zou W. Zhu M.. (2022). New insights into eutrophication management: importance of temperature and water residence time. J. Environ. Sci. 111, 229–239. 10.1016/j.jes.2021.02.033 34949352

[B42] Zhou J. Zheng Y. Hou L. Qi L. Mao T. Yin G.. (2024). Nitrogen input modulates the effects of coastal acidification on nitrification and associated N_2_O emission. Water Res. 261:122041. 10.1016/j.watres.2024.122041 38972235

[B43] Zhu Z. Q. Li X. Bu Q. R. Yan Q. C. Wen L. Q. Chen X. L.. (2023). Land-water transport and sources of nitrogen pollution affecting the structure and function of riverine microbial communities. Environ. Sci. Technol. 57, 2726–2738. 10.1021/acs.est.2c04705 36746765

