# Supplementary material for: Hydrological connectivity shape the nitrogen pollution sources and microbial community structure in a river-lake connected system
Source: Front Microbiol. 2025 Apr 11;16:1563578. doi: 10.3389/fmicb.2025.1563578 (PMC12023755; doi:10.3389/fmicb.2025.1563578)
Supplement: Supplementary file 1 [file Data_Sheet_1.docx]

**Supporting information for**

Hydrological connectivity shape the nitrogen pollution sources and microbial community structure in a river-lake connected system

Haoda Chen^1^, Lulu Zhang*^1, 2^ Zishuai Zheng^1^, Yuang Gao^1^, Yu Zhao*^3^

1. College of Environment Science and Engineering, Hebei University of Science and Technology, 050000, Shijiazhuang, Hebei Province, China
2. Biotechnology Laboratory for Pollution Control in Hebei Province, Shijiazhuang 050018, China
3. State Key Laboratory of Environmental Aquatic Chemistry, Research Center for Eco-Environmental Sciences, Chinese Academy of Science, Beijing, 100085, China

*** Corresponding Authors:**

Lulu Zhang

College of Environment Science and Engineering, Hebei University of Science and Technology, 050000, Shijiazhuang, Hebei Province, China;

E-mail: [zhanglulu19850703@163.com](mailto:zhanglulu19850703@163.com);

Yu Zhao

State Key Laboratory of Environmental Aquatic Chemistry, Research Center for Eco-Environmental Sciences, Chinese Academy of Science, Beijing, 100085, China

1. mail: [yuzhao@rcees.ac.cn;](mailto:yuzhao@rcees.ac.cn;)

**Text S1.** Description of the Baiyangdian Basin

The Baiyangdian Basin is located in the temperate monsoon climate zone, characterized by a warm and humid climate with significant seasonal rainfall. The region experiences an average annual temperature of approximately 12.5°C and an annual precipitation of 500-600 mm. The high-flow period typically occurs from June to September, while the low-flow period extends from December to February. Baiyangdian, the largest freshwater wetland in the North China Plain, is often referred to as the "Kidney of North China," with its water supply primarily dependent on inflows from upstream rivers. The basin covers an area of approximately 4,665 square kilometers and is fed by several tributaries, among which the Fu River, Baigou River, and Xiaoyi River are the main contributors.

The land use patterns and pollution sources within this basin are typical of the region. From upstream to downstream, land use transitions from agricultural areas to densely populated towns and wetland ecosystems. The upstream areas are predominantly agricultural, the midstream passes through several towns and industrial zones, while the downstream is characterized by the wetlands and lakes of Baiyangdian. The pollution types in the Baiyangdian Basin are also broadly representative. Non-point source pollution, primarily from agricultural runoff, rural domestic sewage, and small-scale animal farming, is a major contributor to water quality degradation, with these pollutants often being transported into Baiyangdian via its tributaries. The Fu River basin’s industrial areas and municipal wastewater treatment plants are the main sources of point source pollution.Due to the lack of centralized treatment for rural domestic sewage in the Baiyangdian Basin, septic tanks have become the primary means of rural sewage treatment, with nitrogen pollution from these systems significantly impacting water quality in the basin. Additionally, the scale of animal farming continues to expand, and inadequate waste treatment facilities have exacerbated water pollution issues. This study carefully considers the complex and diverse pollution sources in the Baiyangdian Basin, including agricultural runoff, rural domestic sewage, animal farming waste, industrial effluents, and urban wastewater, making it an ideal case for investigating how pollutant sources and their land-water transport mechanisms affect the ecological processes in lake and river systems.

**Text S2.** Measurement ofphysicochemical parameters

A total of 11 environmental parameters were monitored in this study, including 7 water variables and 4 sediment variables. Nitrate nitrogen (NO_3_^-^-N), ammonium nitrogen (NH_4_^+^-N), total phosphorus (TP), and total nitrogen (TN) were analyzed according to the national standard methods of China (GB7480-87, GB7479-87, GB11893-89, and GB 11894-89, respectively). Sediment characteristics (TP, TN, NH_4_^+^-N, and NO_3_^-^-N) were analyzed based on the criteria established by the Ministry of Ecology and Environment of China (HJ632-2011, HJ717-2014, and HJ634-2012, respectively). All variables were tested in triplicate.

**Text S3.** High-throughput sequencing procedure

PCR amplification was performed in triplicate in a 20μl reaction volume under the following conditions: an initial denaturation at 95°C for 3 minutes, followed by 27 cycles of denaturation at 95°C for 30 seconds, annealing at 55°C for 30 seconds, and extension at 72°C for 45 seconds. A final extension was performed at 72°C for 10 minutes.

For the sequencing data, primers and barcodes exceeding 75% of the amplicon length were removed, and the paired-end reads were merged using Flash software. The merged data were then quality-filtered and demultiplexed using QIIME v1.9.0 software. Operational taxonomic units (OTUs) were classified based on a 97% similarity threshold and compared against the reference database (Silva v128). Taxonomic assignment was performed using the RDP Classifier v2.2, with a classification threshold set at 80%.

**Text S4.** Data sources for SWAT establishment and the sampling principles

Land use data from 2023 were obtained from the Chinese Academy of Sciences and reclassified to align with SWAT model requirements. Soil categories and most physical properties were sourced from the harmonized world soil database, while soil carbon content and available water capacity were derived using the SPAW (Soil-Plant-Atmosphere-Water) platform. Soil chemical properties were obtained from relevant literature and local soil survey reports. A 30-meter resolution Digital Elevation Model (DEM) was utilized to delineate sub-basins. The entire watershed, covering an area of 1,245,647 hectares, was divided into 58 sub-basins with a threshold area of 7,000 hectares, and further subdivided into 3,533 Hydrological Response Units (HRUs) based on homogeneous characteristics of land use, soil type, and slope. Medium- or low-density residential areas (URML), corresponding to rural residential zones, were retained during HRU delineation and used to simulate effluents from rural septic tanks.

Daily records of temperature, precipitation, relative humidity, and wind speed from 2019 to 2023 in the study area were obtained from the China Meteorological Administration.

**Text S5.** Data sources for simulating pollution scenarios in the SWAT model for the Baiyangdian watershed.

In this study, point sources of pollution in the Baiyangdian watershed include industrial zones, industries outside these zones, municipal wastewater treatment plants, rural sewage treatment facilities, concentrated animal feedlot operations, and refuse landfills (Table S1). Nonpoint sources include crop cultivation, scattered small-scale animal feedlot operations, and untreated rural sewage. The total nitrogen emissions from these sources were aggregated for each sub-basin in the Baiyangdian watershed, and their mean monthly loads were input into the SWAT model as point source data.

Specifically, annual nitrogen emissions from industrial zones, industries outside these zones, and concentrated animal feedlot operations were obtained from the Baiyangdian watershed pollution source survey, which was based on government-collected data and field investigations. Additionally, data on annual sewage emissions from municipal wastewater treatment plants, rural sewage treatment facilities, and refuse landfills were collected, and their nitrogen emissions were calculated according to the corresponding discharge standards for each facility. Information on agricultural management schedules specific to the Baiyangdian watershed was gathered through random interviews with local farmers (Table S2).

Data on the rural population within each sub-basin in the Baiyangdian watershed were obtained from local statistical yearbooks. Due to the lack of centralized treatment facilities, rural domestic sewage is mainly managed through conventional septic tanks. The SWAT model's septic tank module was used to simulate rural sewage based on the rural population in each sub-basin. According to previous research, the septic tank effluent flow rate was set at 50 L/day per capita, with total nitrogen (TN) and nitrate-nitrogen (NO₃⁻-N) concentrations of 90 mg/L and 15 mg/L, respectively.

Interview results indicated that most crop fields in the Baiyangdian watershed follow a double-cropping rice rotation, with relatively consistent agricultural management practices across the region.

**Text S6.** SWAT calibration and validation procedure

Daily and monthly flow data from five hydrological stations (Dashuliuzhuang, Xingai, Yaozhuang, Xushui Guoping, and Shifangyuan) for the period 2019 to 2023 were obtained for use in this study.

Monthly observations of TN, NH_4_^+^-N, and NO₃⁻-N concentrations at these stations were gathered over the same period. To estimate monthly nutrient loads, the monthly concentrations of TN, NH_4_^+^-N, and NO₃⁻-Nwere multiplied by the monthly flow values.

**Text S7.** Nitrogen source attribution

Detailed information regarding the nitrogen source attribution procedure can be found in a previous study. Briefly, multiple runs under different pollution input scenarios were conducted to evaluate the nitrogen loads from individual pollution sources. The baseline scenario involved a SWAT run with only fertilizer application, labeled as Loadcrop. The other seven pollution scenarios were designed to investigate nitrogen loadings from various sources. For example, the scenario with both fertilizer application and industrial effluents was labeled as Loadcrop+ind, and the difference between Loadcrop+ind and Loadcrop was interpreted as the nitrogen load attributed to industrial sources. This procedure was repeated to assess the nitrogen loads in the river from different types of point and nonpoint pollution sources.

**Text S8.Quantification Method of River-Lake Hydrological Connectivity**

1.Extracting output data from the SWAT model:

Flow: The flow data for each Hydrological Response Unit (HRU) and subbasin, reflecting the water flow characteristics.

Runoff: The surface runoff data, representing the distribution of water flow within the watershed.

Pollutant load: The pollutant load data from the SWAT model, indicating the distribution of pollutants in different times and spaces.

2.Converting river flow data into a graph model, where the nodes represent different hydrological units, and the edges represent the water flow connections between hydrological units. Based on this, the connectivity (*α*) in graph theory is calculated.

$$\alpha=\frac{\text{Number of edges}}{\text{Total possible edges}}=\frac{E}{N(N-1)/2}$$

*E* is the number of edges in the graph, representing the actual connections between water bodies.

*N* is the number of nodes, representing the number of hydrological units.

Based on the hydrological data from the SWAT model, the river network density and water surface ratio were calculated, reflecting the physical structure of the river system and the extent of water coverage:
River network density(*R*_d_):

$$R_{\text{d}}=\frac{\text{Total river length}}{\text{Total area}}$$

Water surface area (*W*_p_)

$$W_{\text{p}}=\frac{\text{Water surface area}}{\text{Total area}}$$

The interval normalization method was used to standardize each parameter to a range from 0 to 1.

$$X_{\text{i},normalized}=\frac{X_{i}-X_{\min}}{X_{\max}-X_{\min}}$$

$X_{\text{i}}$is original parameter values for each hydrological unit.

$X_{\text{min}}$and$X_{\max}$are the minimum and maximum values of the parameter, respectively.

To comprehensively consider the influence of different parameters on hydrological connectivity, we used the AHP method to assign weights to each standardized parameter.

Finally, we combined the standardized values of each parameter with their respective weights and used the following formula to calculate the Hydrological Connectivity Index $\text{WCI}=\omega_{1}\times\text{Flow}_{\text{normalized}}+\omega_{2}\times\text{Runoff}_{\text{normalized}}+\omega_{3}\times\text{Pollutant load}_{\text{normalized}}$

$\omega_{1}$​,$\omega_{2}$​,$\omega_{3}$​are the weight values determined through the AHP method.

$\text{Flow}_{\text{normalized}}$,$\text{Runoff}_{\text{normalized}}$,$\text{Pollutant load}_{\text{normalized}}$are the standardized values for each parameter.

**Table S1.** Distribution of point sources in the Baiyangdian basin

| **Name of pollution sources** | **Latitude** | **Longitude** | **Industry Classification** |
| --- | --- | --- | --- |
| Lugang Wastewater Treatment Plant (Baoding Drainage Service Center) | 115.4510 | 38.9133 | Municipal Wastewater Treatment |
| Qingyuan Xiangtai Wastewater Treatment Plant (Baoding Runtian Environmental Technology Co., Ltd.) | 115.4925 | 38.7894 | Municipal Wastewater Treatment |
| Xiyuan (Baoding Drainage Service Center - General Discharge - Phase II) | 115.5444 | 38.8644 | Municipal Wastewater Treatment |
| Yindinzhuang Wastewater Treatment Plant | 115.5479 | 38.8646 | Municipal Wastewater Treatment |
| Baoding Zhonghuan Jiacheng Wastewater Treatment Plant | 115.3542 | 38.8167 | Municipal Wastewater Treatment |
| United Environmental Water Services (Gaoyang) Co., Ltd. | 115.8070 | 38.6988 | Municipal Wastewater Treatment |
| Lixian Beiguodan Wastewater Treatment Plant | 115.6886 | 38.6494 | Municipal Wastewater Treatment |
| Anguo Jingmei Wastewater Treatment Co., Ltd. | 115.3333 | 38.4833 | Municipal Wastewater Treatment |
| Lixian Wastewater Treatment Plant | 115.5817 | 38.5072 | Municipal Wastewater Treatment |
| Lixian Xinxing Town Wastewater Treatment Plant | 115.6075 | 38.5703 | Municipal Wastewater Treatment |
| Boye Datong Wastewater Treatment Co., Ltd. | 115.4631 | 38.4286 | Municipal Wastewater Treatment |
| Anguo Jiacheng Wastewater Treatment Co., Ltd. | 115.3333 | 38.4120 | Municipal Wastewater Treatment |
| Dingzhou Zhongcheng Water Services Co., Ltd. | 115.0190 | 38.5016 | Municipal Wastewater Treatment |
| Gezhouba Water Services (Dingzhou) Co., Ltd. | 114.8671 | 38.5238 | Municipal Wastewater Treatment |
| Quyang Datong Wastewater Treatment Plant | 114.7606 | 38.6092 | Municipal Wastewater Treatment |
| Lixian Liushi Town Wastewater Treatment Plant | 115.7491 | 38.5327 | Municipal Wastewater Treatment |
| Qingyuan Dazhuang Town Wastewater Treatment Plant | 115.5071 | 38.6866 | Municipal Wastewater Treatment |
| Dingzhou Qingfengdian Town Wastewater Treatment Plant | 115.0610 | 38.5566 | Municipal Wastewater Treatment |
| Wangdu Qingyuan Drainage Co., Ltd. (Development Zone) | 115.1919 | 38.6480 | Municipal Wastewater Treatment |
| Wangdu Qingyuan Drainage Co., Ltd. (Gaoling Township) | 115.1820 | 38.6988 | Municipal Wastewater Treatment |
| Shunping Qingyuan Wastewater Treatment Plant | 115.1433 | 38.8297 | Municipal Wastewater Treatment |
| Tangxian Wastewater Treatment Plant | 115.0213 | 38.7383 | Municipal Wastewater Treatment |
| Tangxian Changucheng Wastewater Treatment Plant | 114.9950 | 38.7208 | Municipal Wastewater Treatment |
| Baoding Sander Water Treatment Co., Ltd. | 115.4666 | 38.9833 | Municipal Wastewater Treatment |
| Baoding Daceying Water Treatment Co., Ltd. | 115.3447 | 38.9982 | Municipal Wastewater Treatment |
| Baoding Zhongquan Water Services Co., Ltd. | 115.3563 | 38.9678 | Municipal Wastewater Treatment |
| Xushui Dawangdian Town Wastewater Treatment Plant | 115.4758 | 39.0116 | Municipal Wastewater Treatment |
| Baoding Chuangjie Municipal Engineering Co., Ltd. | 115.6566 | 38.9986 | Municipal Wastewater Treatment |
| Dingxing Wuhe Distillery Co., Ltd. | 115.5795 | 39.1399 | Industrial Wastewater Treatment |
| Dingxing Housing and Urban-Rural Development Bureau (Gucheng Town Wastewater Treatment Plant) | 115.7165 | 39.1276 | Municipal Wastewater Treatment |
| Dingxing Jiuxing Agricultural Development Co., Ltd. | 115.6290 | 39.1412 | Agricultural Wastewater Treatment |
| Baigou Town Wastewater Treatment Plant | 116.0225 | 39.1008 | Municipal Wastewater Treatment |
| Gaobeidian City Wastewater Treatment Plant | 115.8965 | 39.3040 | Municipal Wastewater Treatment |
| Dingxing Zhongcheng Poultry Industry Co., Ltd. | 115.8533 | 39.1714 | Agricultural Wastewater Treatment |
| Dingxing Jinrun Textile Printing and Dyeing Co., Ltd. | 115.8892 | 39.2703 | Industrial Wastewater Treatment |
| Dingxing County Wastewater Treatment Plant - Dingxing County Urban Construction Municipal Co., Ltd. | 115.7889 | 39.2156 | Municipal Wastewater Treatment |
| Dingxing Town Wastewater Treatment Plant - Dingxing County Housing and Urban-Rural Development Bureau | 115.7816 | 39.2483 | Municipal Wastewater Treatment |
| Yixian Yuquan Urban Construction Development Co., Ltd. | 115.4993 | 39.3272 | Municipal Wastewater Treatment |
| Laishui Binhe Urban Wastewater Treatment Center (East) | 115.7486 | 39.3908 | Municipal Wastewater Treatment |
| Laishui Binhe Urban Wastewater Treatment Center (West) | 115.6544 | 39.3778 | Municipal Wastewater Treatment |

**Table S2.** Crop management procedures under the wheat-maize rotation.

| Crop | Year | Month | Day | Procedures |
| --- | --- | --- | --- | --- |
| Winter Wheat | 1 | 4 | 20 | Start of the growth season |
| Winter Wheat | 1 | 4 | 20 | Compound fertilizer application (120 kg/ha, basal fertilizer) |
| Winter Wheat | 1 | 6 | 6 | Urea application (60 kg/ha, tillering fertilizer) |
| Winter Wheat | 1 | 6 | 30 | Water release (end of irrigation period) |
| Winter Wheat | 1 | 7 | 12 | Harvest |
| Maize | 1 | 7 | 20 | Sowing and start of growth season |
| Maize | 1 | 7 | 20 | Compound fertilizer application (150 kg/ha, basal fertilizer) |
| Maize | 1 | 8 | 2 | Urea application (80 kg/ha, topdressing) |
| Maize | 1 | 10 | 8 | Water release |
| Maize | 1 | 10 | 22 | Harvest |

**Table S3.** Review of the calibration parameters by variable used by SWAT-CUP

| **Parameter** | **Description** | **Range** | **Calibrated Value** |
| --- | --- | --- | --- |
| CN2.mgt | Curve number for moisture condition II | -0.227385 to -0.163789 | -0.227385 |
| ALPHA_BF.gw | Baseflow alpha factor for groundwater | 0.587098 to 0.73494 | 0.587098 |
| REVAPMN.gw | Threshold depth of water in the shallow aquifer for revap to occur | -298.642395 to -129.728348 | -298.642395 |
| GW_REVAP.gw | Groundwater revap coefficient | -0.536762 to -0.323774 | -0.536762 |
| GWQMN.gw | Threshold water depth in shallow aquifer required for return flow | -0.305401 to -0.245675 | -0.305401 |
| SOL_BD(1).sol | Bulk density of soil layer | 0.113505 to 0.358781 | 0.113505 |
| CH_K2.rte | Effective hydraulic conductivity in main channel | 61.810043 to 158.820602 | 61.810043 |
| CH_N2.rte | Manning's "n" value for main channel | 0.070693 to 0.150009 | 0.070693 |
| GW_DELAY.gw | Groundwater delay time | 26.179174 to 49.137875 | 26.179174 |
| ESCO.hru | Soil evaporation compensation factor | 0.740373 to 0.871827 | 0.740373 |
| EPCO.hru | Plant uptake compensation factor | 0.769135 to 1.016485 | 0.769135 |
| CANMX.hru | Maximum canopy storage | 0.261864 to 0.406756 | 0.261864 |
| SMFMN.bsn | Minimum melt rate for snow | -21.083895 to -14.533034 | -21.083895 |
| SOL_AWC(1).sol | Available water capacity of soil layer | -0.724089 to -0.633337 | -0.724089 |
| TIMP.bsn | Snow pack temperature lag factor | -0.319392 to -0.00985 | -0.319392 |
| OV_N.hru | Manning's "n" value for overland flow | -0.214462 to -0.091726 | -0.214462 |
| SLSUBBSN.hru | Average slope length | 0.011894 to 0.100282 | 0.011894 |
| HRU_SLP.hru | Average slope of HRUs | -0.71607 to -0.558492 | -0.71607 |
| SOL_K(1).sol | Saturated hydraulic conductivity of soil layer | -0.708197 to -0.517445 | -0.708197 |
| SFTMP.bsn | Snowfall temperature | -5.83032 to -5.157282 | -5.83032 |
| SURLAG.bsn | Surface runoff lag coefficient | 1.019251 to 1.208369 | 1.019251 |
| SPCON.bsn | Linear parameter for channel sediment routing | 0.002088 to 0.003108 | 0.002088 |
| SPEXP.bsn | Exponent parameter for channel sediment routing | 1.771576 to 1.918158 | 1.771576 |
| USLE_P.mgt | USLE support practice factor | 0.205009 to 0.309585 | 0.205009 |
| BIOMIX.mgt | Biological mixing efficiency | 0.580056 to 1.144494 | 0.580056 |
| RSDCO.bsn | Residue decomposition coefficient | -0.000922 to 0.044884 | -0.000922 |
| ERORGN.hru | Organic N enrichment ratio | 0.512294 to 4.41083 | 0.512294 |
| SDNCO.bsn | Denitrification threshold water content | 0.360954 to 0.65338 | 0.360954 |
| NPERCO.bsn | Nitrogen percolation coefficient | -0.131061 to 0.432779 | -0.131061 |
| BC1.swq | Rate constant for biological oxidation of NH₄⁺ to NO₂⁻ | 0.480728 to 0.738214 | 0.480728 |
| BC2.swq | Rate constant for biological oxidation of NO₂⁻ to NO₃⁻ | -0.324727 to 0.771867 | -0.324727 |
| BC3.swq | Rate constant for hydrolysis of organic N to NH₄⁺ | 0.057636 to 0.220542 | 0.057636 |
| PHOSKD.bsn | Phosphorus partitioning coefficient | 192.89035 to 285.15799 | 192.89035 |
| PPERCO.bsn | Phosphorus percolation coefficient | 7.509212 to 12.602797 | 7.509212 |
| AI2.wwq | Fraction of algae biomass that settles | 0.016989 to 0.021993 | 0.016989 |
| SOL_ORGP.chm | Organic P content in soil layer | 48.517494 to 90.48156 | 48.517494 |
| PSP.bsn | P availability index | -0.013416 to 0.553792 | -0.013416 |
| ERORGP.hru | Organic P enrichment ratio | -1.605889 to 1.647271 | -1.605889 |
| RS5.swq | Settling rate of organic N in the reach | 0.002507 to 0.088449 | 0.002507 |

**Table S4.** The abundance of the populations of eight biomarkers in the RF analysis

| Phylum | OTU numbers | Relative abundance |
| --- | --- | --- |
| Actinobacteriota | 359092 | 0.161280355 |
| Patescibacteria | 17419 | 0.007823462 |
| Deferrisomatota | 267 | 0.000119919 |
| GAL15 | 3808 | 0.001710302 |
| NB1-j | 3477 | 0.001561638 |
| FCPU426 | 60 | 2.69E-05 |
| Synergistota | 3018 | 0.001355486 |
| Chloroflexi | 350426 | 0.157388161 |

**Table S5.** Nitrogen and Oxygen Isotope Endmember Values (Mean ± SD) of Potential Nitrate Sources Used in the SIAR Model

| Source | Sampling Time | δ^15^N (‰) Mean ± SD | δ^18^O (‰) Mean ± SD |
| --- | --- | --- | --- |
| Atmospheric Deposition (AP) | May-23 | 4.58 ± 8.24 | 29.0 ± 20.96 |
| Chemical Fertilizer (CF) | May-23 | 13.04 ± 4.4 | 6.6 ± 3.79 |
| Soil Nitrogen (SN) | May-23 | 7.73 ± 4.87 | 24.54 ± 28.75 |
| Manure and Sewage (M&S) | May-23 | 15.92 ± 12.43 | 17.56 ± 15.71 |
| Atmospheric Deposition (AP) | Aug-23 | 4.93 ± 3.91 | -3.92 ± 2.68 |
| Chemical Fertilizer (CF) | Aug-23 | 10.2 ± 3.69 | -0.85 ± 1.89 |
| Soil Nitrogen (SN) | Aug-23 | 7.96 ± 3.64 | 4.4 ± 1.44 |
| Manure and Sewage (M&S) | Aug-23 | 9.94 ± 6.59 | -0.74 ± 1.83 |


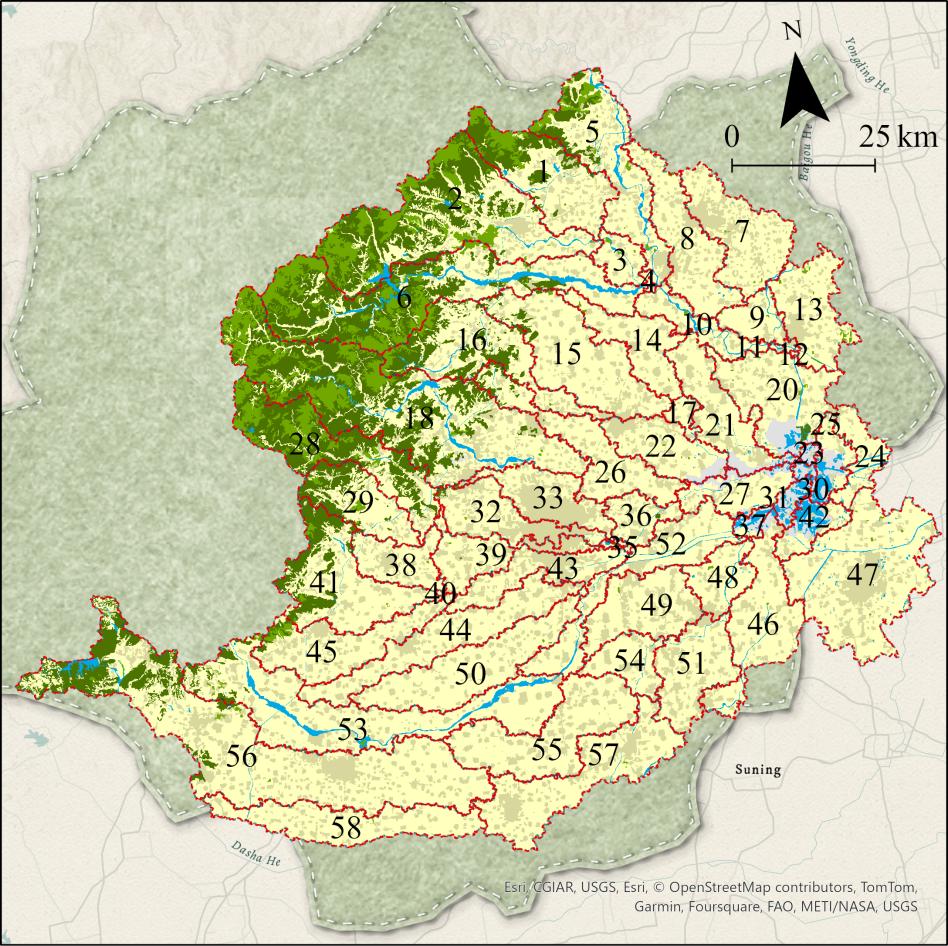


**Figure S1.** Integration of Data for SWAT Model, Including Land Use, Soil Characteristics, DEM, Meteorological Information, and Monthly Monitoring of Nitrogen Species


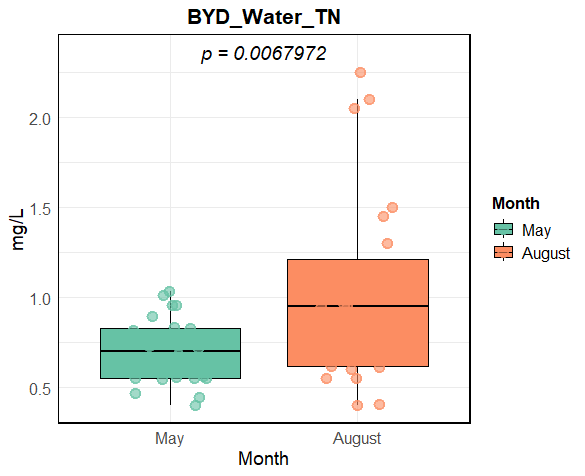

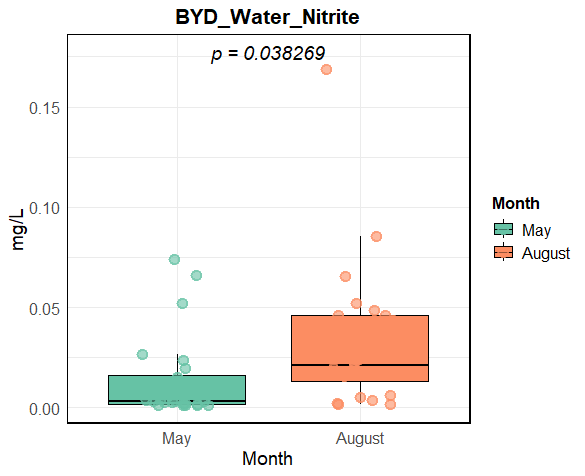

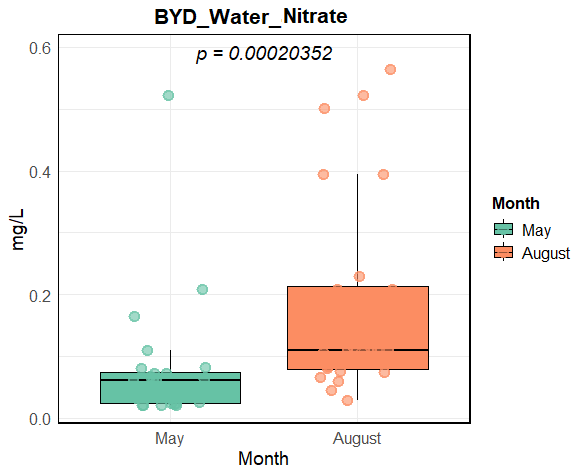

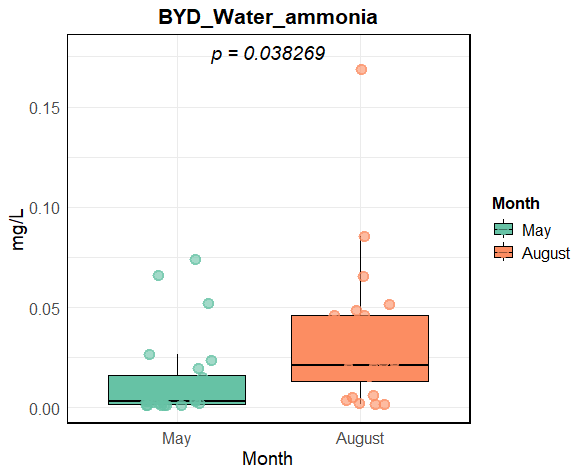

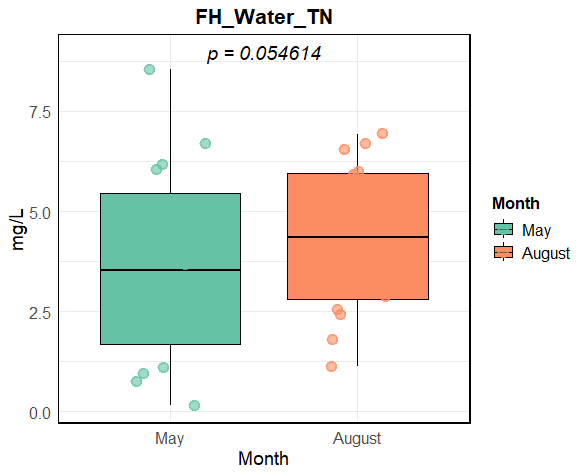

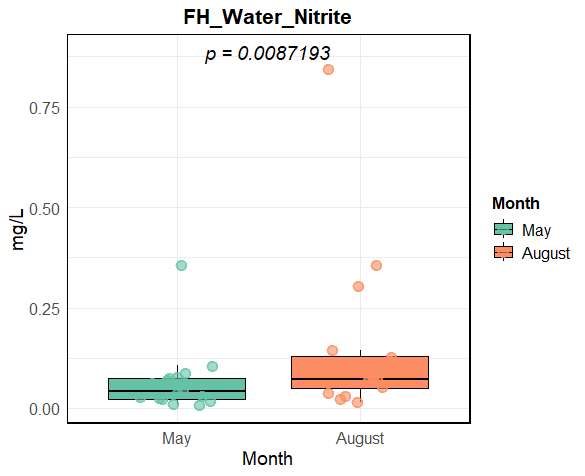

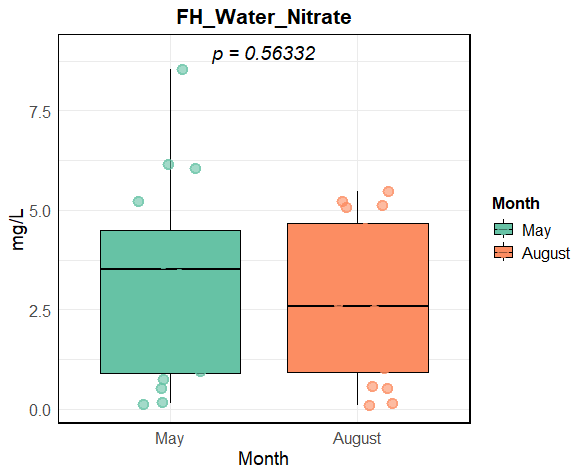

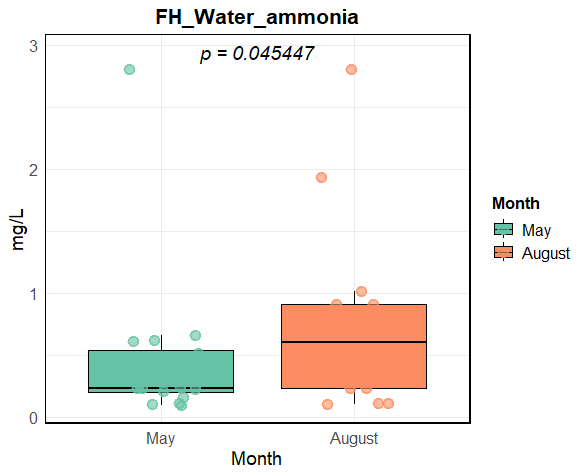

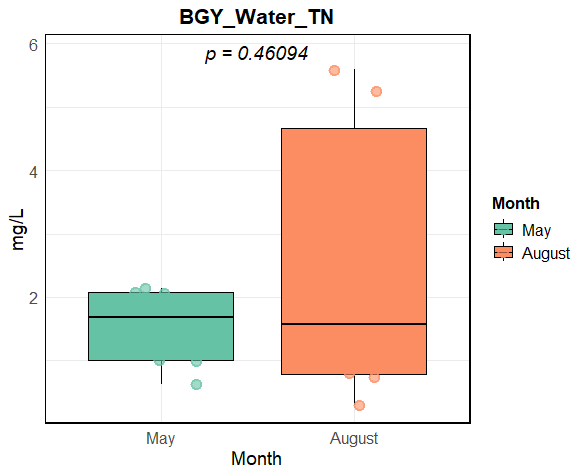

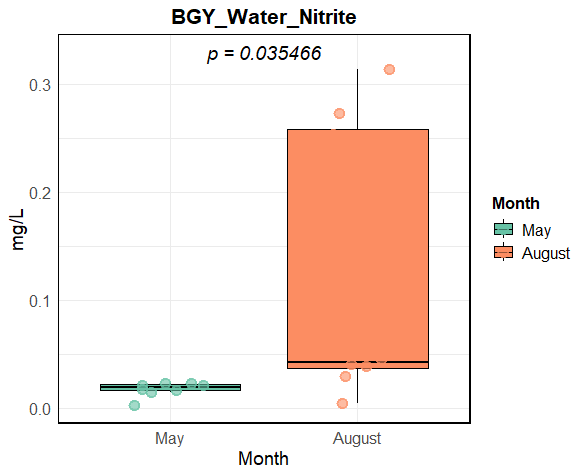

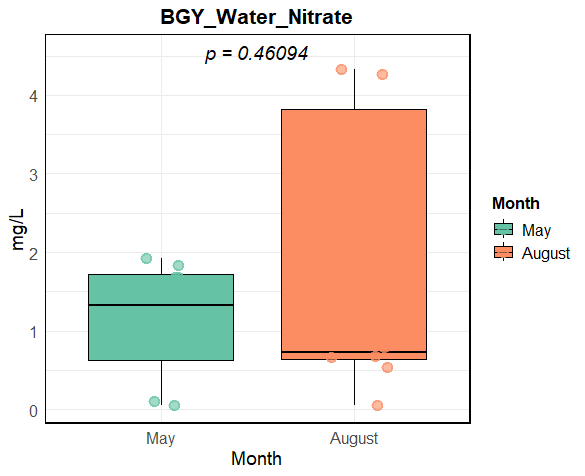

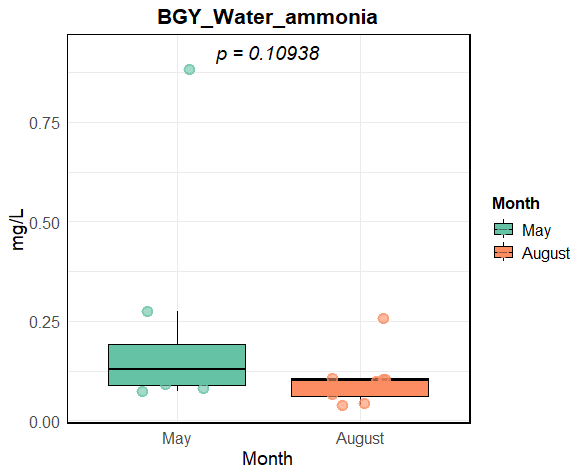

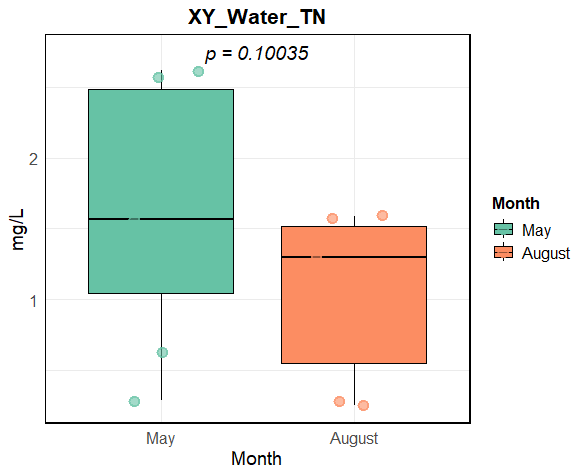

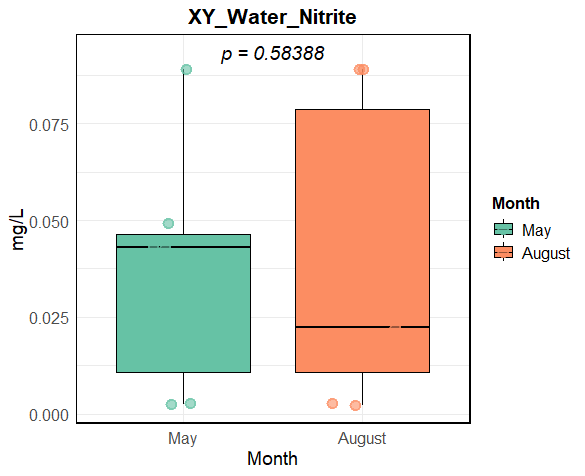

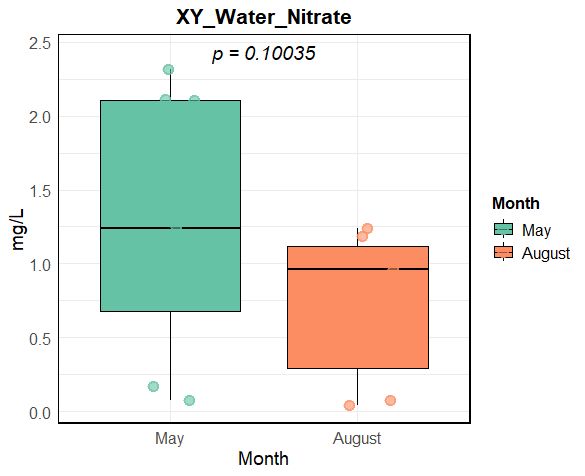

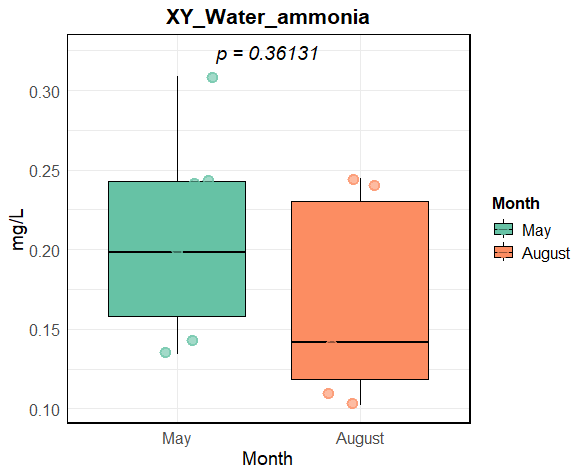


**Figure S2.** Comparison of the physicochemical properties of water at sampling locations among the BYD, HF, BGY, and XY groups


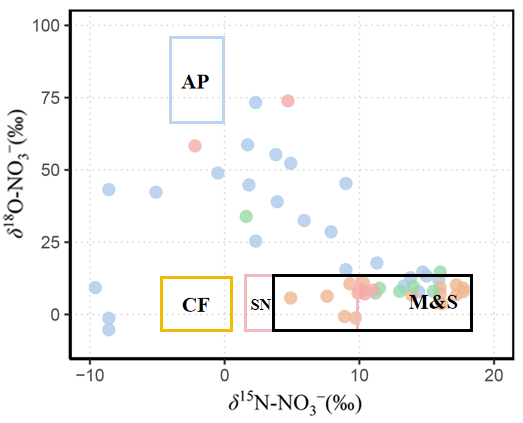

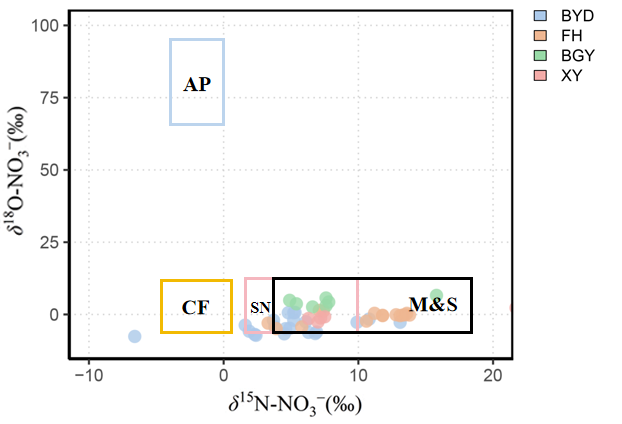


**Figure S3.** Isotopic compositions of δ^15^N-NO_3_^-^ and δ^18^O-NO_3_^-^ for surface water samples together with typical nitrate source regions for atmospheric deposition (AP), chemical fertilizer (CF), soil nitrogen (SN), and manure&sewage (M&S). The δ^15^N values for MS are adapted from measurements within the Baiyangdian Basin while the other sources were measured from local pollution sources. δ^18^O values for AP were measured from local precipi­ tation samples and the other sources were calculated using nitrification theory


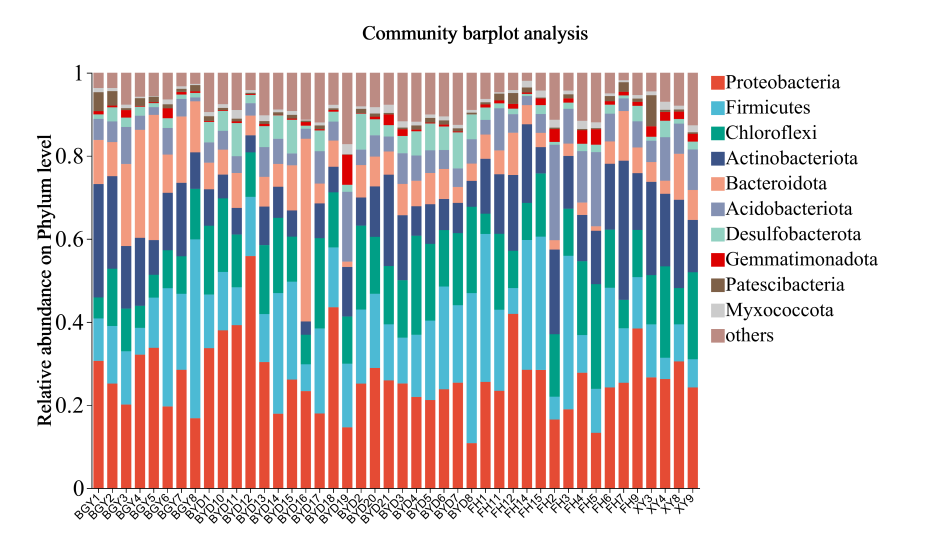

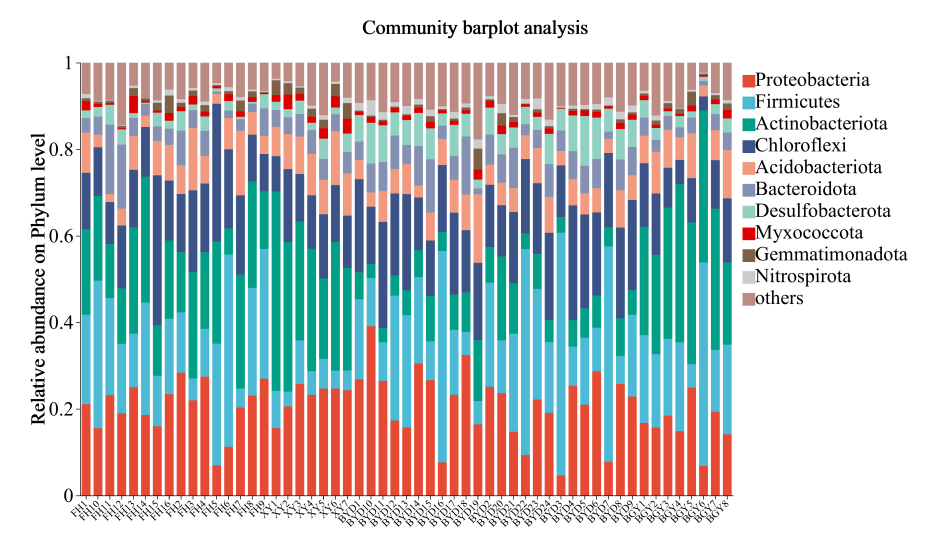


**Figure S4.** Microbial Community Composition in May and August at Different Sampling Sites


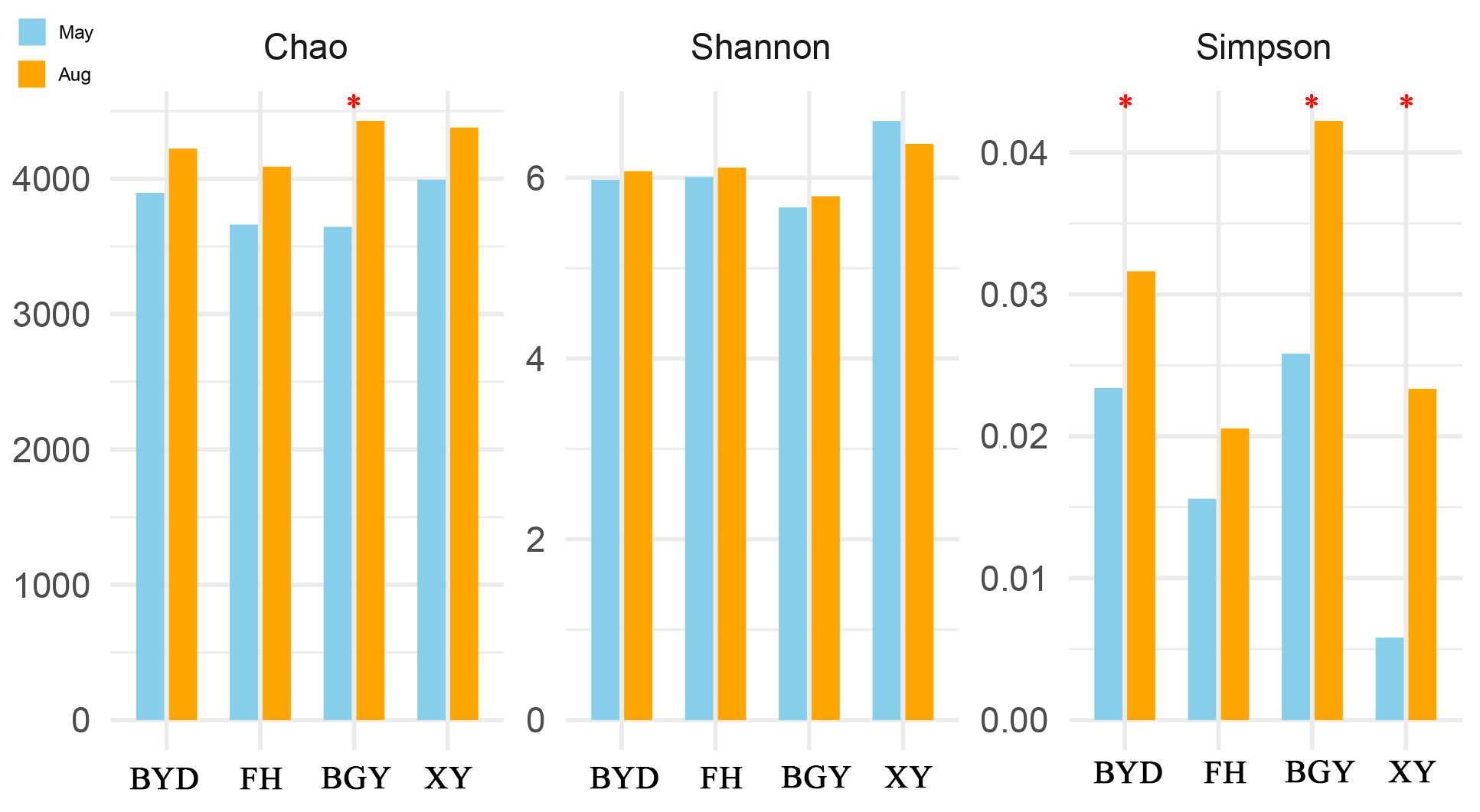


**Figure S5.** Alpha diversity indices (Chao1, Shannon, Simpson) of microbial communities in May and August


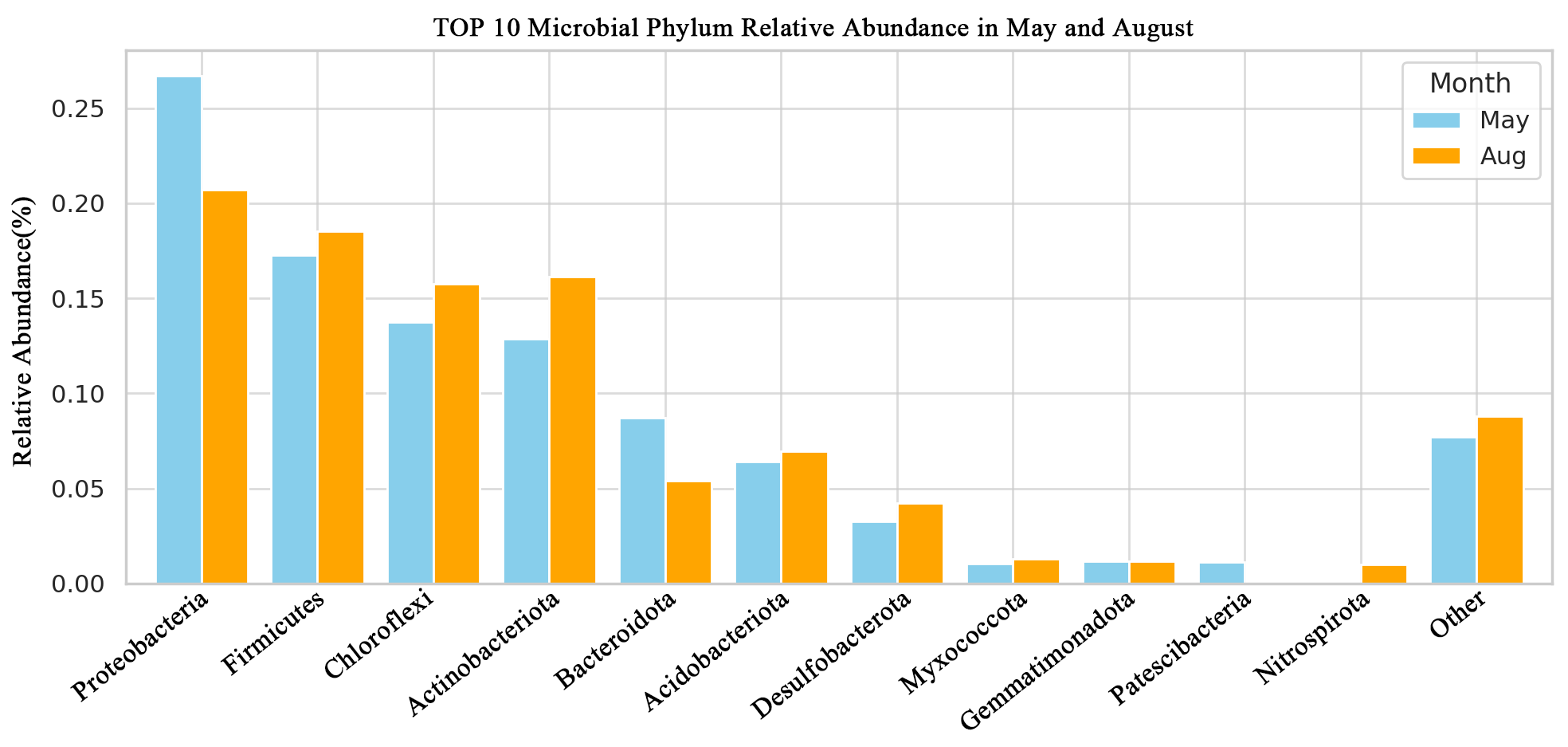
**Figure S6.**The relative abundance of the top 10 microbial phyla observed in May and August


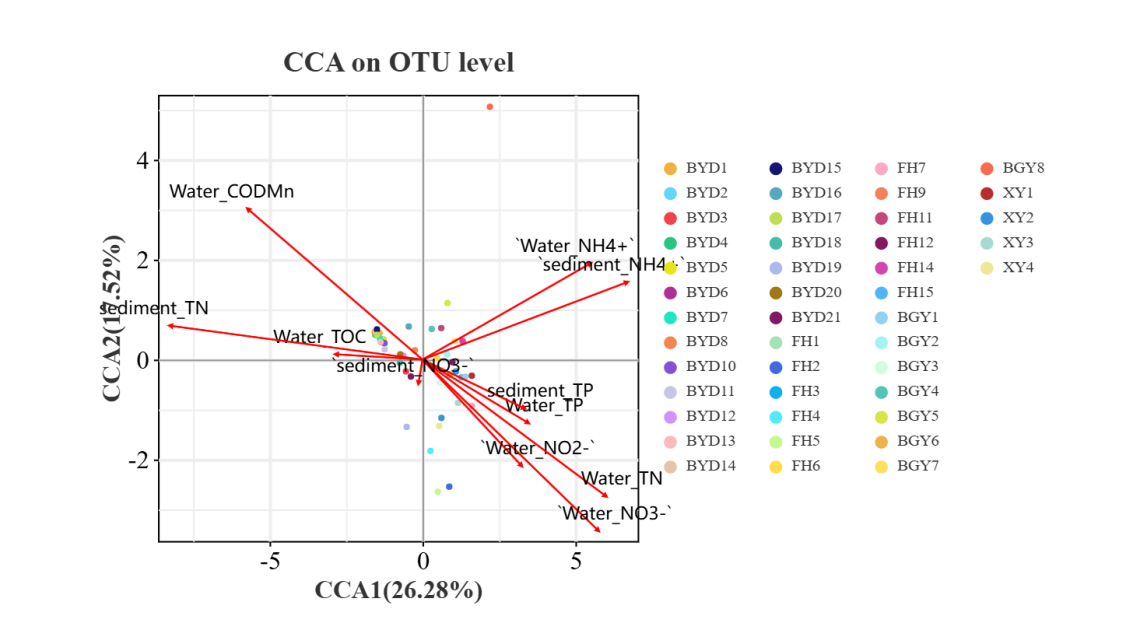

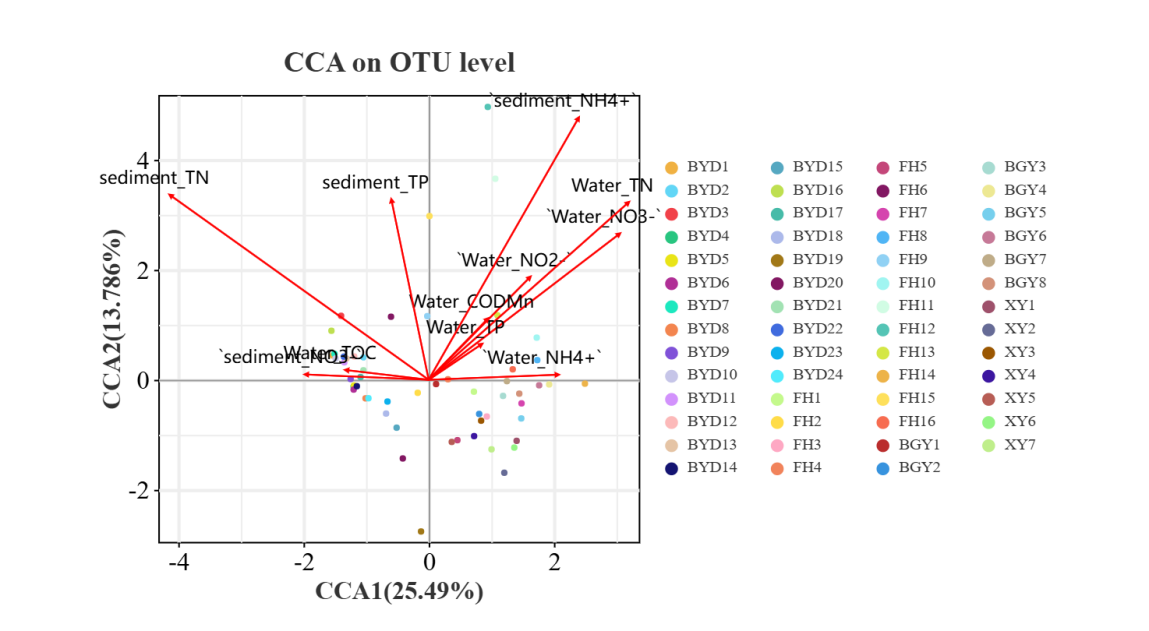


**Figure S7.** Canonical Correspondence Analysis (CCA) on OTU Level for Environmental Factors in May and August.


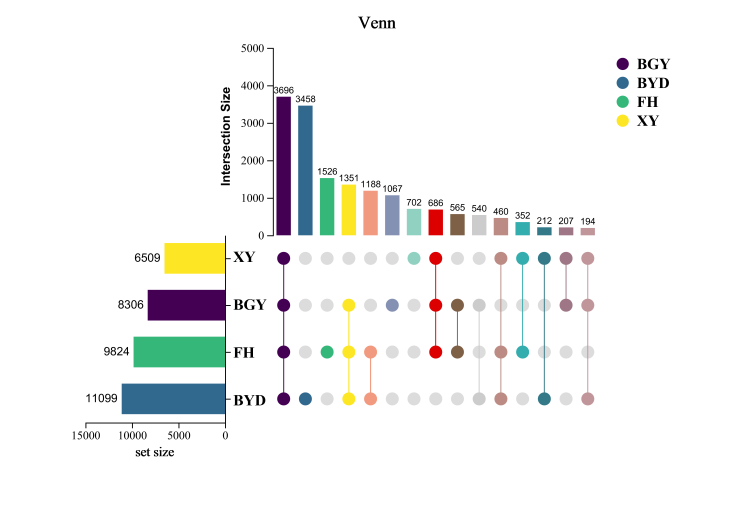

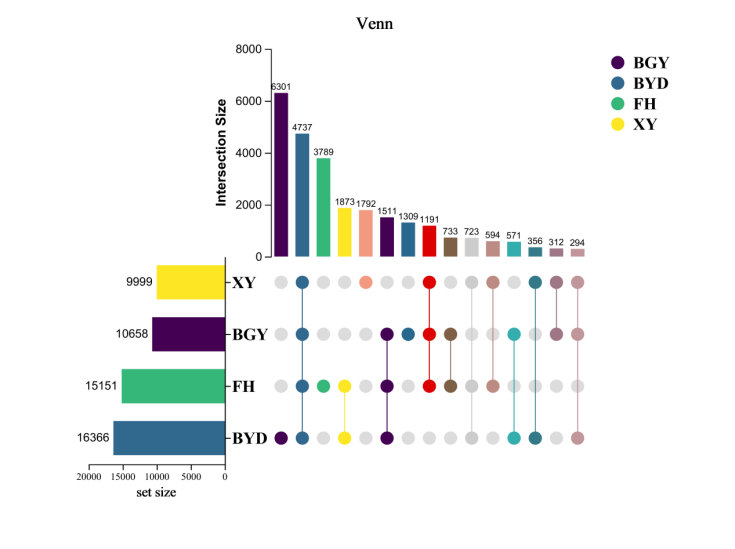


**Figure S8**. Comparison of the numbers of species between BGY group, BYD group, FH group and XY group.


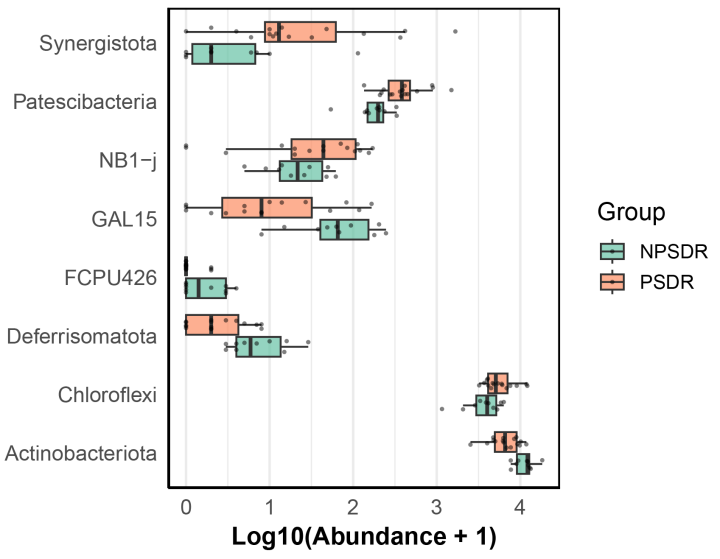


**Figure S9.** The relative proportions of biomarkers in the BGY group, the BYD group, the FH group and the XY group.


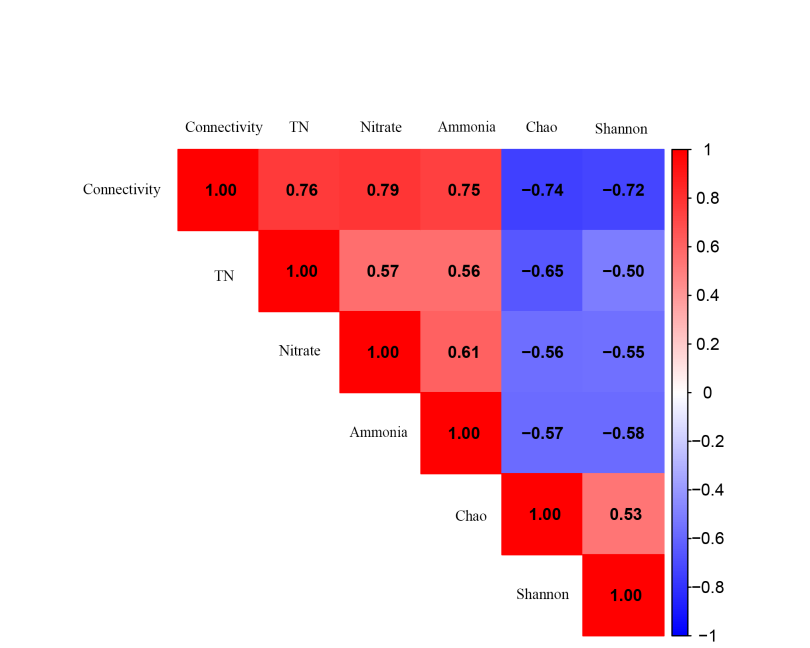


**Figure S10.** Pearson correlation matrix showing relationships between River-Lake Connectivity, nitrogen concentrations (TN, Nitrate, Ammonia), and microbial alpha diversity indices (Chao1, Shannon)
